# Generation of Phenothiazine with Potent Anti-TLK1 Activity for Prostate Cancer Therapy

**DOI:** 10.1016/j.isci.2020.101474

**Published:** 2020-08-20

**Authors:** Vibha Singh, Siddhant Bhoir, Rupesh V. Chikhale, Javeena Hussain, Donard Dwyer, Richard A. Bryce, Sivapriya Kirubakaran, Arrigo De Benedetti

**Affiliations:** 1 Department of Biochemistry and Molecular Biology, LSU Health Sciences Center, Shreveport, USA; 2 Department of Biological Engineering, Indian Institute of Technology Gandhinagar, Gandhinagar, India; 3 Division of Pharmacy & Optometry, University of Manchester, Manchester, UK; 4 Department of Chemistry, Indian Institute of Technology Gandhinagar, Gandhinagar, India; 5 Department of Psychiatry and Behavioral Medicine, LSU Health Sciences Center, Shreveport, USA

**Keywords:** Medical Biochemistry, Structural Biology, Cancer

## Abstract

Through *in vitro* kinase assays and docking studies, we report the synthesis and biological evaluation of a phenothiazine analog J54 with potent TLK1 inhibitory activity for prostate cancer (PCa) therapy. Most PCa deaths result from progressive failure in standard androgen deprivation therapy (ADT), leading to metastatic castration-resistant PCa. Treatments that can suppress the conversion to mCRPC have high potential to be rapidly implemented in the clinics. ADT results in increased expression of TLK1B, a key kinase upstream of NEK1 and ATR and mediating the DNA damage response that typically results in temporary cell-cycle arrest of androgen-responsive PCa cells, whereas its abrogation leads to apoptosis. We studied J54 as a potent inhibitor of this axis and as a mediator of apoptosis *in vitro* and in LNCaP xenografts, which has potential for clinical investigation in combination with ADT. J54 has low affinity for the dopamine receptor in modeling and competition studies and weak detrimental behavioral effects in mice and *C. elegans.*

## Highlights

• J54, a safe and potent inhibitor of TLK1, was generated via *in silico* docking studies
• J54 leads to apoptosis of PCa cells in combination with anti-androgen administration
• J54 has low affinity for dopamine receptor and low anti-dopaminergic activity in animals
• TLK1B is selectively increased upon ADT thereby providing a specific target for PCa

## Graphical Abstract

**Figure:**
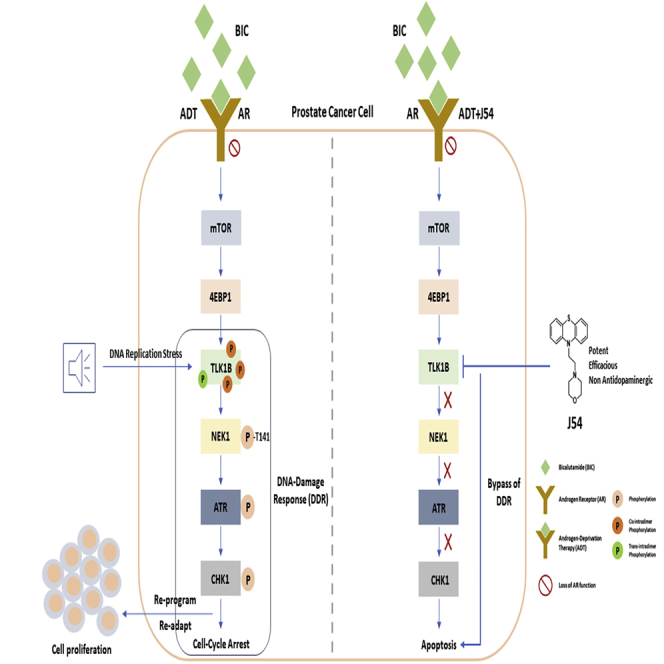

## Introduction

Prostate cancer (PCa) is a leading cause of morbidity and mortality of men in the western world. The standard of care for advanced PCa after failure of localized treatments is androgen deprivation therapy (ADT) and anti-androgens (Karantanos et al., 2013; Tao et al., 2019), which provides respite from disease progression, but ultimately fails resulting in the incurable phase of the disease: mCRPC. Treatments that can suppress the conversion to mCRPC have the best potential to improve outcome and be rapidly implemented in the clinics, and this requires a clear understanding of the process of PCa cells' mechanisms of adaptation to ADT. This process has been well studied in the LNCaP cell line model, in which we have recently elucidated some missing details (Singh et al., 2019a). Androgen deprivation of LNCaP cells results in loss of AR function with a compensatory pro-survival activation of mammalian target of rapamycin (mTOR) (Carver et al., 2011) and concomitant implementation of cell division arrest by activation of the DNA damage response (DDR) mediated by ATR-Chk1 (Chiu et al., 2012) or ATM-Chk2 (Reddy et al., 2015). The DDR is likely activated due to the role played by the AR as replication licensing factor (Litvinov et al., 2006) in combination with the mTOR-dependent increased expression of TLK1B, which is highly dependent on the mTOR>4EBP1 pathway (Sunavala-Dossabhoy et al., 2004), and resulting activation of the Nek1>ATR > Chk1 axis (Singh et al., 2019a). Additional work from our laboratory suggested that this may be a conserved nexus in additional cell models, in the TRAMP mouse, and probably in many patients, as the specific activating phosphorylation of Nek1 by TLK1 correlates with the Gleason score (Singh et al., 2019b). The resulting cell-cycle arrest is a survival mechanism for PCa cells, which remain quiescent until they reprogram and adapt to androgen-independent (AI) growth. An attractive strategy to prevent this process would be to bypass the cell-cycle arrest via inhibition of ATM or ATR, causing the cells to undertake replication with damaged DNA that would cause mitotic catastrophe, a strategy that was in fact implemented in LNCaP treated concomitantly with bicalutamide (BIC) and ATM inhibition (Reddy et al., 2015). However, a limitation of this approach is how to make the inhibition of ATM or ATR specific to PCa cells to limit general toxicity. We have recently demonstrated that addition of a relatively specific inhibitor of TLK, thioridazine (THD) (Ronald et al., 2013), which we repurposed for the blockage of the axis, results in fact in apoptosis of LNCaP and TRAMP-C2 cells concomitantly treated with BIC. In addition, it suppresses the late re-growth of PCa in the TRAMP mouse following castration (Singh et al., 2019a, 2019b). However, THD is a known anti-psychotic and has undesirable side effects. Here we describe J54, a potent and safe TLK1 inhibitor, as an adjuvant to ADT for PCa.

## Results

### A Next-Generation PTH as a Potent Inhibitor of TLK1B

**Figure 1:**
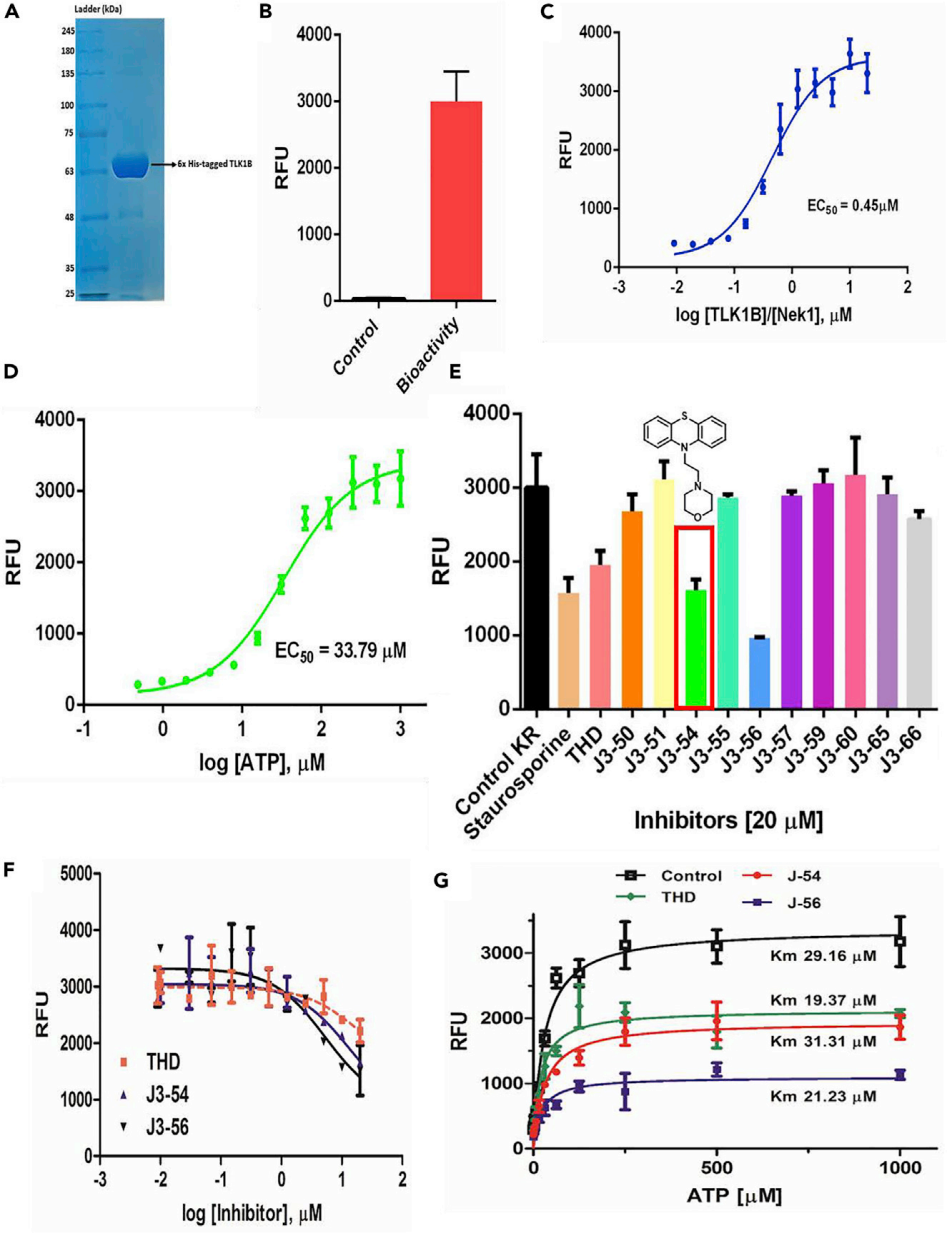
Kinase Assays, Inhibitor Screening, IC_50_ Evaluation, and Competitive Assays (A–C) Recombinant TLK1B purified to homogeneity (A) is active in kinase assays (B) and phosphorylates a specific Nek1 peptide (C). (D) ATP dependence. (E) Various compounds were tested for *in vitro* inhibitory effects, with J54 shown. (F and G) Inhibitory curves of J54 and J56 compared to THD were carried out at 0.1 mM ATP (F) or at different competitive concentrations (G). The data presented are the mean ± SE of n = 3 independent experiments. The DiscoverRx ADP Hunter (Eurofins DiscoverRx, Fremont, CA, USA) Kit was used to measure the generation of ADP resulting from kinase phosphorylation of substrate. Further details are explained in Section C of Supplementary Material Methods.

A few PTH anti-psychotics were identified in a compounds library screen as good inhibitors of TLK1 (Ronald et al., 2013). To find additional inhibitors, recombinant TLK1B was purified as previously described (Bhoir et al., 2018) (Figure 1A). The enzymatic activity was determined using the ADP-Hunter reagents and a Nek1 peptide containing the T141 target site. Properties of substrate (Nek1 peptide) and ATP dependence were determined (Figures 1B–1D), and in subsequent reactions with inhibitors used at 0.1 mM, which were found to be optimal. Several structurally similar compounds were synthesized as described in Supplemental Information (see NMR and mass spectrometry data Figures S3 and S4; and also Data S2) and tested at 20 μM (Figures 1E and 1F). J3-54 (thereafter J54) and J3-56, both being PTH derivatives, were as or more inhibitory than staurosporine, which is the standard pan-kinase inhibitor. We continued our characterization of J54. The other synthesized compounds did not show much inhibition, even though several are PTH (see Table S1). We have carried out some structure-activity relationship studies and molecular modeling to explain why, and for simplicity, we show below a comparison between THD and J54.

### *In Silico* Modeling Studies of TLK1B with Docked J54 or THD

**Figure 2:**
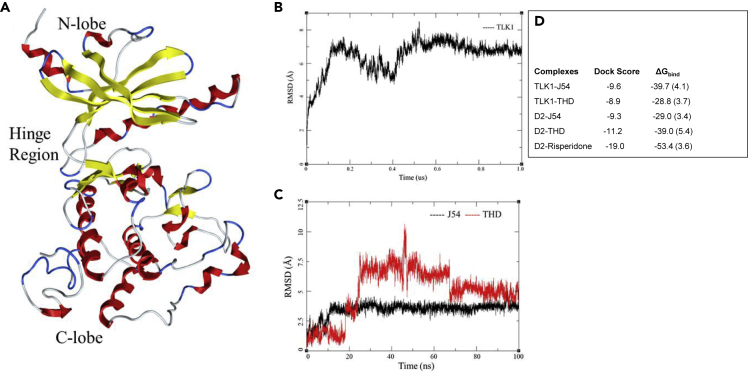
Model Building and Molecular Dynamics Studies (A) Model of TLK1B kinase domain. (B) Protein RMSD for TLK1B over 1-μs simulation. (C) Ligand RMSD of J54 (black) and THD (red) in post-docking simulation for 100 ns. (D) Docking score and computed free energy for predicted pose of ligand bound to TLK1 or D2 receptor proteins. Standard deviations in parenthesis. Energies in Kcal/mol.

**Figure 3:**
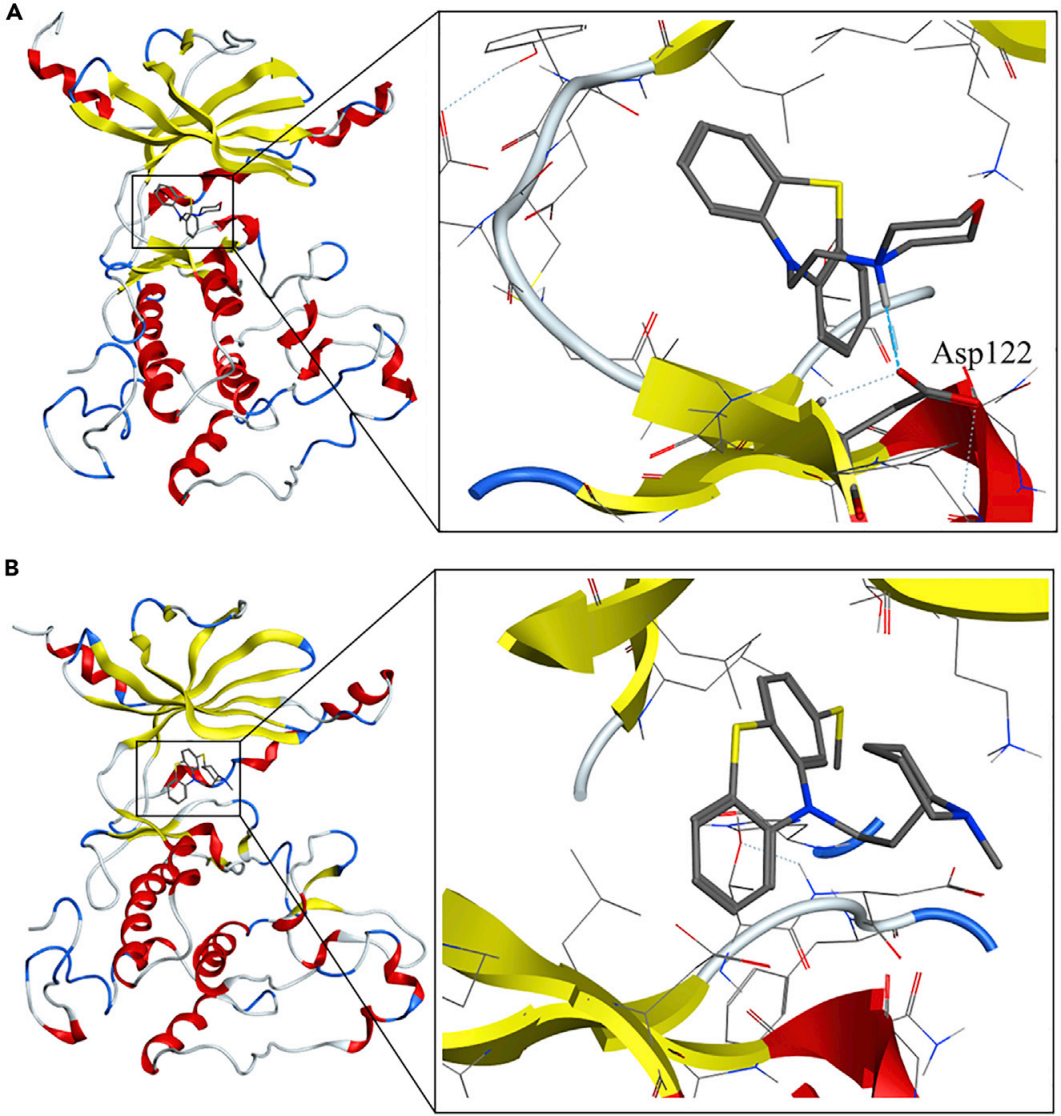
Molecular Docking and Molecular Dynamics Studies of J54 and THD with TLK1 (A) Interactions of J54 with the active site of TLK1. (B) Interactions of THD with the active site of TLK1.

A model of the TLK1B kinase domain was constructed by homology modeling using the ROBETTA *de novo* protein structure prediction server (Song et al., 2013) (see Transparent Methods in Supplemental Information) and further compared/aligned to the published crystal structure of the highly homologous TLK2 (Mortuza et al., 2018). A PDB file for the structure was included as Data S1. The model was further refined by a 1-μs molecular dynamics (MD). The backbone root-mean square deviation (RMSD) of the structure stabilized after 100 ns, although, was subject to periodic RMSD shifts until 600 ns (Figure 2A). Compounds J54 and THD were docked into the ATP site of the final MD-refined model (Figures 2B and 2C). In the docked pose of compound J54, good interactions with the hinge region residues of TLK1 were exhibited; the morpholino head forms hydrogen bonds with Asp122, with a distance of 1.68 Å from the Asp122 carboxylate Oδ to the ligand NH group (Figure 3A). A 100-ns MD simulation of this TLK1-J54 complex indicated that the pose is stable, further evident from a favorable total binding free energy ΔG_bind_ = −39.7 kcal/mol, computed by the MM/GBSA method (Genheden and Ryde, 2015) (Figure 2D). THD was docked in the same TLK1 pocket but did not form hydrogen bonds with the protein (Figures 3B and S1). MD of the complex indicated a markedly higher RMSD in ligand atoms, with greater fluctuation, than for the J45 complex (Figure 2C); the free energy of binding is 9.9 kcal/mol weaker (Figure 2D). We note that this in part could arise from the methylthio group of THD, which prevents it from entering the hinge region to the same extent as J54 (Figure S1).

### J54 in Combination with Anti-androgen (Bicalutamide) Induces Apoptosis in AS PCa Cells

**Figure 4:**
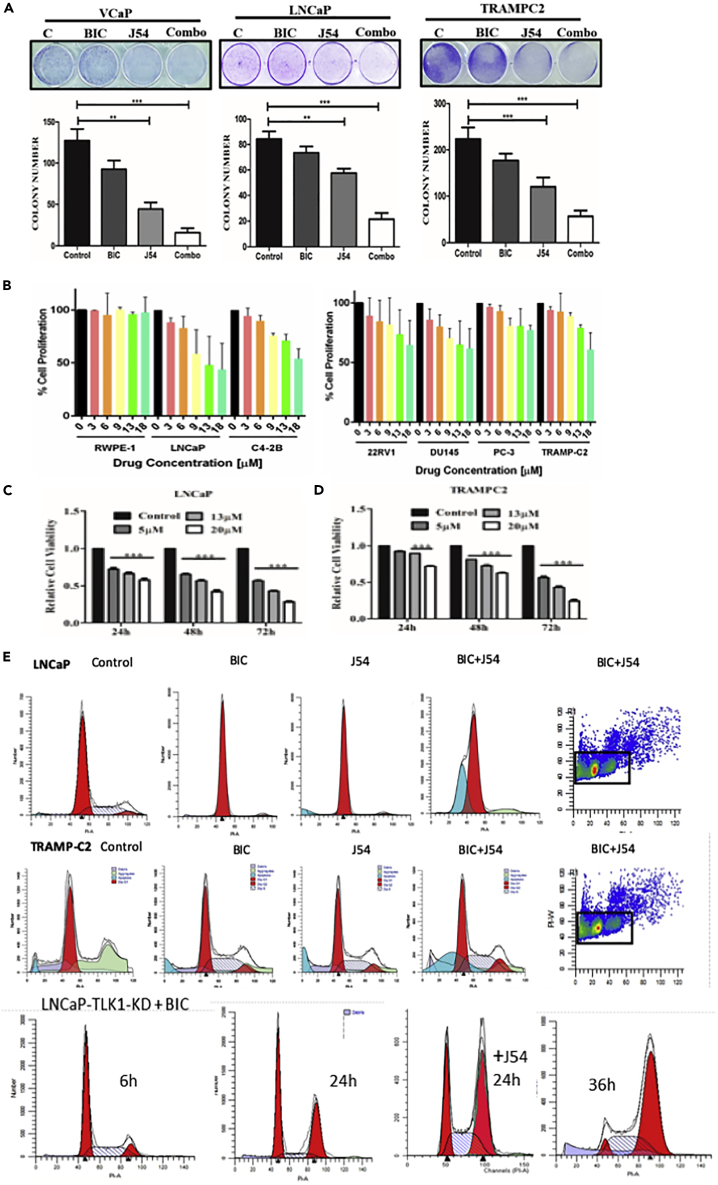
J54 in Combination with an Anti-androgen (Bicalutamide) Induces Apoptosis in AS PCa Cells All experiments were conducted in triplicates. (A) Clonogenic assays of AS PCa cells (VCaP, LNCaP, and TRAMP-C2) after treatment with BIC, J54, or combination. The cells were grown for 2–3 weeks and stained with crystal violet. All experiments were conducted in triplicates. Two-way ANOVA tests were done to compare the group for statistical significance; ∗∗ p < 0.01, ∗∗∗ p < 0.001. (B) Cell proliferation assays of the indicated cell lines incubated with different concentrations of J54. The cell lines used were human “normal” RWPE-1, LNCaP, (LNCaP-derivative) C4-2B, 22RV1, DU14, and PC3 and mouse TRAMP-C2. All experiments were conducted in triplicates, and two-way ANOVA tests were done to compare the group for statistical significance. (C and D) Cell proliferation of LNCaP (C) and TRAMP-C2 (D) were determined during 3-day incubation with different concentration of J54 (MTS assay). Two-way ANOVA tests were done to compare the group for statistical significance; ∗∗p < 0.01, ∗∗∗p < 0.001. All experiments were conducted in triplicates. (E) Cell cycle analysis by PI-FACS of LNCaP and TRAMP-C2 cells incubated for 24 h with BIC, J54, or combination (5 μM each). Representative analysis of two independent experiments are shown. LNCaP cells overexpressing dominant-negative TLK1-KD treated with BIC accumulate in G2 before dying over the next few days.

**Figure 5:**
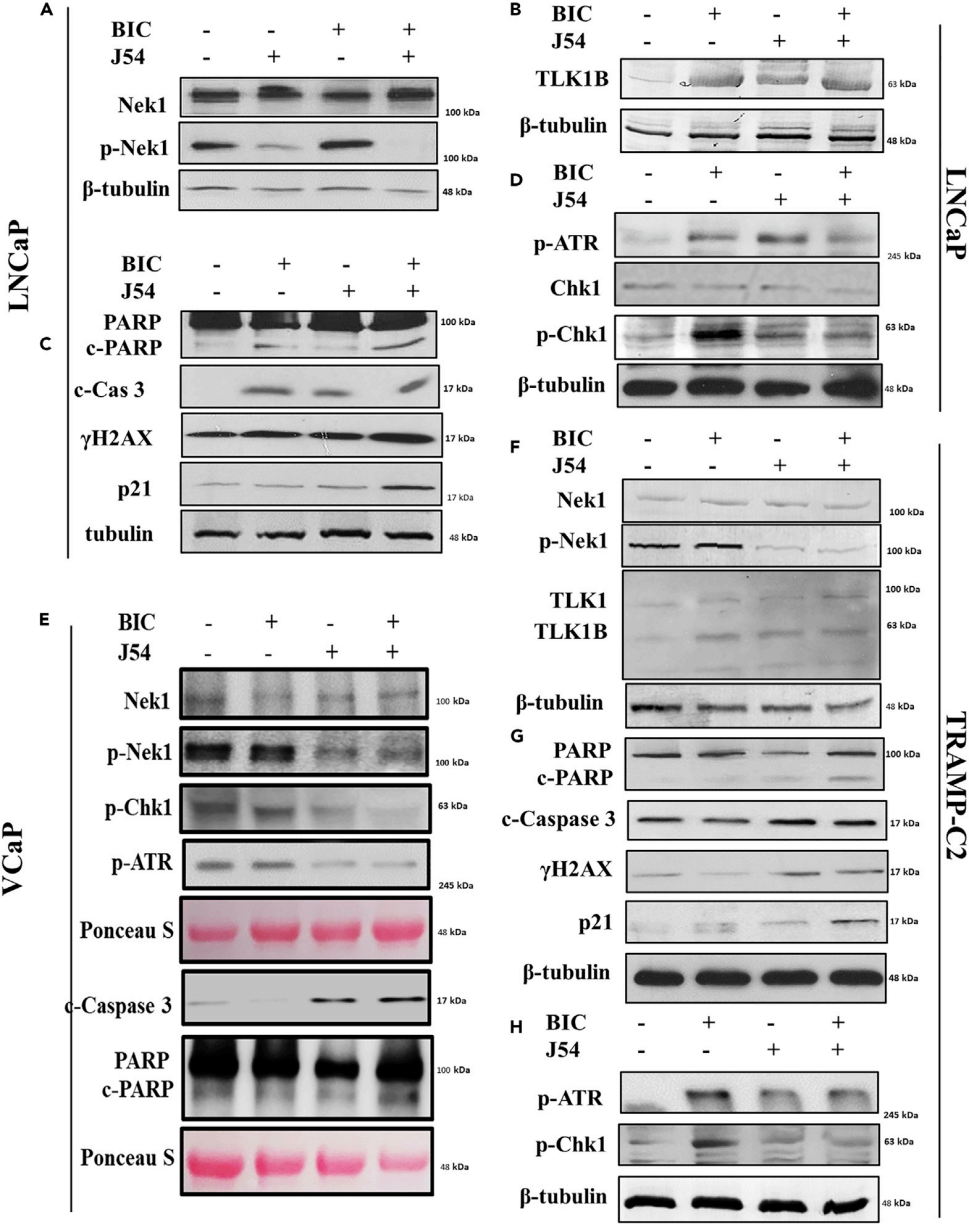
J54 in Combination with Bicalutamide Suppresses the Checkpoint Activation Markers and Induces Apoptotic Markers Western blots (WB) of cell cycle and apoptotic indicators. (A–D) LNCaP, (E) VCaP, and (F–G) TRAMP-C2 cells from the four treatment groups as indicated were analyzed by WB for several indicators of DNA damage/apoptosis and mediators of cell-cycle arrest. Representative analysis of two independent experiments for each cell line is shown.

We previously reported that THD has some growth inhibitory effects for several PCa cells lines, either androgen sensitive (AS) or insensitive (AI) (Singh et al., 2019a). We thus tested the same panel of cells in androgen-containing medium (FBS) by proliferation assays. The effect was a weak dose-dependent inhibition with maximal efficacy around 18 μM (Figure 4B). Note, however, that RWPE1 (normal prostate) cell line was insensitive to J54, suggesting that J54 may be a more targeted anti-cancer agent, or it is possible that the TLK1>Nek1 axis remains critical even for CRPC cells. In contrast to these mild effects, combination treatment of the AS cell lines, LNCaP, VCaP, and TRAMP-C2, with BIC and J54 resulted in 4- to 5-fold suppression in colony formation (Figure 4A, p = 0.001). Note that VCaP cells were 60% inhibited by J54 alone, consistent with their elevated replication stress-driven DDR and checkpoint activation (Figure 5E), which when suppressed with J54 could lead to apoptosis. Clonogenic assays are unable to distinguish the effects of a DNA damaging agent, which can result in either an impaired or delayed resumption of growth (cell division) or loss of viability due to increased killing of the initial population. We thus measured in LNCaP and TRAMP-C2 cells the early change in cell number (MTT assay) over 72 h with J54 alone. The results indicated an actual loss in cell counts in relation to the dose (Figures 4C and 4D, p = 0.001). These results reinforced our main thesis that inhibiting the TLK1>Nek1 axis is mostly an effective regimen for AS cells in combination with anti-androgens. Indeed, cell cycle analysis of LNCaP and TRAMP-C2 cells treated with BIC, J54, or combination for 24 h showed a strong increase in the fraction of apoptotic cells only in the combination group (Figure 4E). Note also that, unlike TRAMP-C2 treated with BIC that display an accumulation of cells at G1/S and reduction of the S and G2 populations, cells treated with both BIC and J54 do not display the arrest (particularly no loss of the G2 cells), i.e., bypass of the G1 checkpoint and consequent apoptosis (Table S2). However, note that J54 treatment of LNCaP cells resulted in a modest reduction of the S-phase cells, so we cannot exclude that in some PCa cells J54 may cause a G1 arrest. To confirm these results and the specificity of the TLK1 inhibition with a genetic approach that could obviate potential off-target effects of J54, one possibility would be to deplete TLK1, but this is problematic because of the redundant function of TLK2. However, an alternative is to overexpress a dominant kinase-dead TLK1 (TLK1-KD) because this protein can inhibit both TLK1 and TLK2 as these kinases homo- and hetero-dimerize for full activation (Mortuza et al., 2018). In fact, we have reported that overexpression of TLK1-KD efficiently suppresses pNek1-T141 (Singh et al., 2017). We have generated LNCaP cells overexpressing TLK1-KD and found that after treatment with BIC alone, the cells fail to form AI colonies after 2 weeks and instead die. Interestingly, cell cycle analysis of these cells during a time course after addition of BIC showed an accumulation of cells in G2 before they begin to die, and not G1 as parental LNCaP (Figure 4E). In many cell types, including LNCaP, accumulation of cells in G2 is an indication of DNA damage post-replication, consistent with our interpretation of an important role of TLKs in mediating a G1/S checkpoint and preventing accumulation of incompletely replicated DNA (due to loss of AR activity). In fact, it was reported that TLKs stabilize replication forks (Lee et al., 2018), although these authors have not reported an effect for TLKs' knockdown in suppressing ATR activation. However, they showed that ATM was not activated in cells depleted of TLK1-2, until they added UCN-01, a specific Chk1 inhibitor (Lee et al., 2018). This is consistent with our interpretation that bypassing the Chk1-mediated arrest in cells deprived of androgen and depleted of TLK activity results in replication fork collapse and activation of ATM. The apparent difference between suppression of TLK function by overexpressing TLK1-KD versus chemical inhibition with J54 can be explained by the more rapid induction of apoptosis for the latter, thus masking the G2 accumulation. When the TLK1-KD overexpressing cells were treated with BIC + J54 for 24 h (before apoptosis) the proportion of cells arrested in G2 was greater than the proportion of cells incubated with BIC alone. However, we have not tested the activation of ATR/Chk1 and ATM/Chk2 in these cells to better asses these central mediators of the DDR and cell cycle checkpoints. This is therefore suggested evidence that TLKs are important for implementing the DDR and cell-cycle arrest after ADT and that bypassing this role results in apoptosis instead of leading to AI adaptation.

### J54 with Bicalutamide Suppresses the Checkpoint Markers and Induces Apoptotic Markers

LNCaP, VCaP, and TRAMP-C2 are the main examples of established PCa cell lines that are initially AS, but when maintained in ADT condition, recapitulate the conversion to AI growth observed in patients, and start growing again. In the initial phase of ADT, they arrest cell division, primarily at the G1/S transition, and become quiescent, which is a protective effect from undergoing replication fork collapse upon inhibition of the licensing function of the AR (Litvinov et al., 2006). As inhibition of the TLK1B > Nek1>ATR > Chk1 axis with THD results in bypass of the DDR and apoptosis (Singh et al., 2019a), we have tested the same process with J54. First, we confirmed that J54 (with or without BIC) causes a reduction in the p-Nek1-T141, the site of activation that is phosphorylated by TLK1, in all three AS cell lines (Figures 5A–5H); another established TLK1 substrate, Rad9-S328, also was less phosphorylated after addition of J54 (Figure S7D). We were also able to reproduce that the expression of TLK1B was induced in LNCaP and TRAMP-C2 following the addition of BIC, in contrast to the expression of the TLK1 splice variant that remains unchanged (Figure 5F). We previously showed that the increase in TLK1B is due to an mRNA translational effect caused by the compensatory increase in mTOR activity following ADT and suppression of AR signaling (Carver et al., 2011); note that there was no change in the TLK1B mRNA with BIC (Figure S7B). J54 also resulted in a futile increase inTLK1B, and not TLK1, possibly via a selective drug-induced stabilization of TLK1B, which turns over fairly rapidly (unpublished observations). Treatment with BIC resulted in activation of the DDR, shown as an increase in p-ATR and p-Chk1 in LNCaP and TRAMP-C2. In contrast, suppression of p-Nek1 and its activity with J54 resulted in inhibition of the BIC-induced DDR activation (Singh et al., 2017, 2019a, 2019b), as manifested by a decrease in p-ATR and p-Chk1 (Figures 5A–5H). J54 alone caused a modest increase in p-ATR but not in p-Chk1 in LNCaP and TRAMP-C2, as we previously observed with THD (Singh et al., 2017), possibly due to a mild genotoxic effect. The VCaP cells were a little different because even without any BIC, p-ATR, and p–Chk1 were already elevated. This has been observed before (Karanika et al., 2017) and attributed to a constitutive DDR activation due to the TMPRSS2-ERG fusion in these cells (Chatterjee et al., 2015). Nonetheless, the addition of J54 to VCaP cells resulted in reduction of p-Nek1 and p-Chk1, indicating that it can suppress the DDR checkpoint, whether activated constitutively or after BIC treatment (Figure 5E). As we previously reported for inhibition of TLK1 with THD (Singh et al., 2019a), bypass of the BIC-induced checkpoint can result in generation of DSBs (indicated by the presence of γH2AX) and leads to apoptosis (indicated by increased cleaved caspase 3 and PARP, Figures 5A–5H). Likewise, in BIC + J54-treated cells there was an increase in P21 expression (Figures 5C and 5G), an indicator of the emergency activation of the P53 > p21 pathway, and an effect previously observed for BIC + THD-treated LNCaP and TRAMP-C2 cells that resulted in pATM-S1981 activation from generation of DSBs (Singh et al., 2019a) and reproduced for J54 + BIC (Figure S7C). The induction of p21 has been likewise reported in TRAMP-C2 cells treated with imiquimod and correlated with accumulation of cells in G2 before the onset of apoptosis (Han et al., 2013).

### Bicalutamide with J54 Suppresses Growth of LNCaP Xenografts via Suppression of the TLK1B >pNek1 DDR Pathway and Promotes Apoptosis

**Figure 6:**
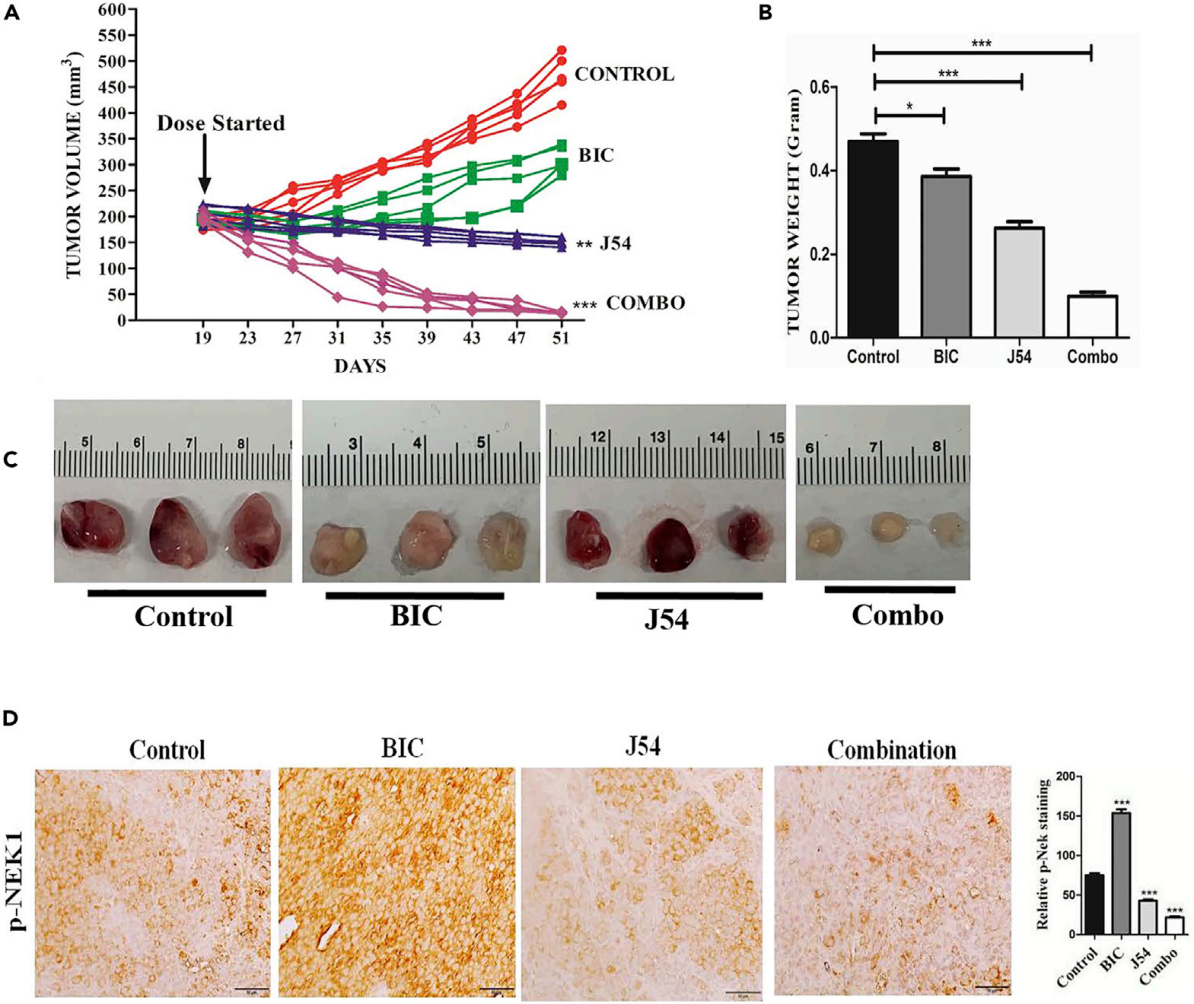
Combination Bicalutamide and J54 Suppresses Growth of LNCaP Xenografts (A) Time course of tumor growth of LNCaP cells xenografts in 4 treatment groups. Treatment started 19 days after implantation when the tumors measure ~200 mm^3^. Two independent experiments with 5 mice per group were carried out. J54 and bicalutamide were dissolved in DMSO and diluted in corn oil 1:10 and administered intraperitoneally biweekly. Sectioning and processing of the tissues were carried out in the FWCC Histology Service, using automated processes and equipment to provide uniform and standardized results. Indirect labeling was with ABC Elite: RTU Vectastain Elite Reagent, Vector #PK-7100; DAB: ImmPact DAB, Vector #SK-4105. Light counterstaining was done with hematoxylin. (B) Tumor weights were determined for all groups at end of the treatment course. (C) Examples of tumor size at the end of the experiment. (D) Representative sections from tumors resected from mice in the 4 treatment groups analyzed by immunohistochemistry for pNek1 (n = 3 mice; scale bar, 50 μm). Note the weak pNek1 stain in the tumor from mice treated with J54, in contrast to the increase seen with BIC. Two-way ANOVA tests were done to compare the group for statistical significance; ∗p < 0.05, ∗∗p < 0.01, ∗∗∗p < 0.001.

We sought to establish if the addition of J54 could suppress the resurgence of tumor growth of LNCaP xenografts and their later conversion to CRPC. Following formation of sizable tumors (∼200 mm^3^), castration, or anti-androgens arrest the progression of LNCaP xenografts for some time (2–3 weeks), whereas subsequently the tumors start growing again at an accelerated rate, which is refractory to ADT (AI). Therefore, we injected LNCaP cells in Matrigel in both flanks of NOD-SCID mice and then randomly assigned them to four treatment groups (n = 5 per group × 2 independent experiments), as shown in Figures 6A–6C. The control group showed progressive exponential growth, and so did the BIC group after a 12-day lag following the beginning of BIC administration. Interestingly, treatment with J54 alone showed significant suppression of tumor growth (p = 0.01) and tumor weight (p = 0.001), whereas the combination (BIC + J54) resulted in complete suppression of tumor growth and weight (p = 0.001) and actual regression of the tumors compared with the starting size Figures 6A–6C. An immunohistochemical analysis of the available excised tumors showed that the phosphorylation of Nek1-T141 was increased in BIC-treated group (Figure 6D, p = 0.001), consistent with a corresponding increase in TLK1B expression (Singh et al., 2019a, 2019b); it was suppressed by concomitant administration of J54 (p = 0.001), which is expected to result in bypass of the DDR checkpoint and increased apoptosis and corresponding markers. In fact, the combination treatment showed a strong increase in staining for cleaved PARP and caspase 3, and γH2AX (a marker of DNA damage, p = 0.001, Figure S8). Tumors in the combination also showed a reduction in the number of proliferative cells by Ki67 staining (p = 0.001), although this was lower also for the groups singly treated with BIC and J54 (Figure S8, note that these tumors were isolated at day 30 when they were still sizable). We should also point out that we treated the mice with J54 only biweekly due to the favorable pharmacokinetics, where maximal plasma concentration of 100 ng/mL (35 μM) was reached 2 h after intraperitoneal injection, and was still present at ∼6 ng/mL (6 μM) after 24 h (Figures S5A–S5C).

### J54 Has Low DR2 Binding Activity and Behavioral Effects in Animal Models

THD has strong anti-tumor effects in combination with ADT for AS-PCa models (Singh et al., 2019a, 2019b), but after ∼30 years of use, this drug was withdrawn for treatment of schizophrenia due to increased risk of cardiac arrhythmia (Wu et al., 2015) and extrapyramidal toxicity. However, we reasoned that other PTH inhibitors of TLK1 could have similar benefits for the treatment of PCa without the side effects. The repurposing of PTH anti-psychotics for cancer therapy has been proposed (Lee et al., 2016), even though their cellular targets have not been identified, as they were generally assumed to work largely through inhibition of dopamine receptors (Yin et al., 2015). Interestingly, an analysis from five independent studies of PCa incidence in individuals with schizophrenia revealed a significant decrease in standardized incidence ratio ranging from 0.49 to 0.76 (95% confidence interval) (Torrey, 2006) (Mortensen, 1989). This reasoning has led to the design and synthesis of J54. However, these observations beg the question of whether J54 has also anti-dopaminergic activity. We thus considered the potential interaction with the D2 dopamine receptor (DR2). Compounds J54, THD, and DR2 antagonist risperidone were studied by molecular docking, MD, and free energy calculations, based on the risperidone/DR2 crystal structure (PDB code 6CM4) (Wang et al., 2018). The three ligands were docked in the DR2 pocket (docking was able to reproduce the observed pose of risperidone). The three complexes were solvated and simulated via MD for 100 ns. Compound J54 binds stably in the active site of the DR2, forming a hydrogen bond with Ser159 distance of 2.24 Å (Figure S2A, related to Figure 2D). THD and risperidone also complex with the DR2 via hydrogen bonds (Figures S2A and S2C, related to Figure 2D). Interestingly, the binding free energy computed via MM/GBSA ranks J54 as the lowest affinity ligand, with a ΔG_bind_ value of −29.0 (Figure 2D). This is followed by THD (−39.0 kcal/mol) and then risperidone (−53.4 kcal/mol). These free energy calculations suggest that J54 binds only weakly and has a lower affinity than risperidone and THD toward DR2. To verify experimentally that J54 is a weak DR2 antagonist, we commissioned from Sekisui-Xenotech a competitive radioassay using 7-hydroxy DPAT, R-(+)-[3H] as a tracer, and two recombinant human dopamine receptors (D1 and D3). Positive control antagonists were included [R(+)-SCH-23390 and (±)-7-Hydroxy-2-(di-n-propylamino) tetralin ((±)-7-OH-DPAT)]. In Table S3, related to Figure 2D, we show the results obtained at effective concentrations of 100 nM for J54 compared with THD, which confirmed that J54 is a very weak antagonist for dopamine receptors. Note that in these competitive studies with recombinant receptors, the antagonist (e.g., THD) is typically active in the 10–100 nM range (IC50, 20 nM for THD), whereas for DR2 antagonism for amelioration of psychotic conditions, circulating concentrations in plasma need to be ∼100 μM.

We then did a general assessment of mice following injections of J54. We noticed no toxicity (even after gross inspection of organs at necropsy), no decrease in body weight, and no behavioral changes (no lethargy or extrapyramidal twitches sometime observed with THD). To get a better assessment of the possible behavioral effects of J54, we used *C. elegans* that has a simple but complete nervous system and has been well characterized for its responses to anti-psychotic drugs, including actions at serotonin or dopamine receptors (Donohoe et al., 2006, 2009). Consistent with the weak binding of J54 to DR2 (Figure 2D), in three studies, J54 had much weaker behavioral effects then TFP or risperidone, attributable to DR2 activity (Figures S6A–S6C). Note that the concentration of these drugs on agar plates need to be significantly higher than in tissue culture medium for mammalian cells because of the protective and poorly permeable cuticle in *C. elegans.*

## Discussion

Certain PTH are potent inhibitors of TLK1 (Ronald et al., 2013), and by inference TLK2, as these proteins must homo- and hetero-dimerize for full activation (Mortuza et al., 2018), in addition to being highly similar in sequence and structure, and thus similarly targetable. In fact, a Kinomescan carried out at 15 μM with J54 resulted in inhibition of TLK1 and TLK2 and little inhibition of all other kinases with the exception of TTK (“SCANMAX” file, Table S4, related to inhibition data in Figure 1). This was similar to what we previously found for THD and PPH (Ronald et al., 2013) providing additional evidence for the specificity of PTH compounds with respect to other kinases except perhaps for reported inhibition of PKC (Aftab et al., 1991) that was not tested here as it is not included in the SCANMAX list, which also does not include key PIKK (see Limitations). Two noteworthy observations are that PTHs are structurally different from many known ATP competitive inhibitors, and that J54 was carefully designed based on considerations reported previously for the THD—the Kinomescan has confirmed that J54 is specific to TLKs. We should point out that although PTH were the first reported inhibitors of TLKs (Ronald et al., 2013), other inhibitors particularly of TLK2 have since been reported (Kim et al., 2016), some based on structure docking (Mortuza et al., 2018), although neither their potency and effects *in vivo* nor their potential off-target effects have not been reported as they were selected from libraries of kinase inhibitors.

As not all PTH are good inhibitors of TLK1 (Ronald et al., 2013 and this study), it was critical to first sort out *in silico* which compounds best fit in modeled binding site. We have now shown that J54 has higher affinity than THD by MD analysis, and hence is more potent and likely more specific for TLK1. We have previously reported that a TLK1 inhibitor, THD, could synergize with ADT in promoting apoptosis on AS PCa cell lines in culture and in xenografts (Singh et al., 2019a), and in the TRAMP mouse model (Singh et al., 2019b). This rationale was derived from our previous discovery that TLK1 is an upstream activator of the Nek1>ATR > Chk1 axis [11], in conjunction with an original result that showed that TLK1B is translationally increased following AR suppression (Singh et al., 2019a) and the established compensatory mTOR activation (Carver et al., 2011). Abrogation of the TLK1>Nek1>ATR axis was expected to result in bypass of the DDR checkpoint and thus promote mitotic catastrophe. This was also consistent with the known role of AR signaling in regulation of DNA repair in PCa and synergistic killing with inhibitors of DNA repair (Karanika et al., 2017; Thompson et al., 2017)

It seems clear that certain PTH anti-psychotics correlate with a decreased risk of PCa development (Torrey, 2006), whereas the use of THD and some other PTH anti-psychotics presents some risks and side effects. Therefore, we have developed a second-generation TLK inhibitor that has lower affinity for the DR2. The potent dopamine antagonist activity of some PTH antipsychotics has been blamed for some of the lethargic and extrapyramidal effects, as well as for the cardiac arrhythmia. *In silico*, J54 showed much weaker binding to the DR2 than THD or risperidone, and it appeared to have little adverse behavioral effects in mice and worms.

In this work, several PCa cell lines were studied for growth inhibition by J54, and particularly the AS cell lines, LNCaP, VCaP, and TRAMP-C2, were sensitive to apoptosis when combined with an anti-androgen (BIC). The LNCaP model was also tested in xenografts and demonstrated remarkable tumor regression. In conclusion, we suggest the use of J54 as adjuvant therapy for PCa in conjunction with anti-androgens, as a safer and more potent inhibitor of this DDR axis, which we believe is commonly activated during the initial phase of PCa cells' adaptation to ADT. In conclusion, the regulation of the DDR by TLK1 through the Nek1>ATR > Chk1 axis, and even more importantly its upregulation after ADT, is an important finding in the field of PCa research and as a target for potential therapy (Singh et al., 2017, 2019a, 2019b). A very large amount of work in PCa therapy has been devoted to the search for better anti-androgens, whereas relatively little has been devoted to combining ADT with targeting the known role of the AR in controlling the DDR (Karanika et al., 2015; Thompson et al., 2017). Our approach, which may seem to go counter-current to the established views of standard of care for advanced PCa, is to abrogate the ADT-induced DDR checkpoint and cell-cycle arrest, thereby forcing apoptosis of PCa cells still responsive to ADT. Although it seems clear that certain PTH anti-psychotics correlate with a decreased risk of PCa development (Torrey, 2006), the use of THD and some other PTH anti-psychotics presents some risks and side effects. In particular, an increased risk for cardiac arrhythmia (Hennessy et al., 2002) as a result of their anti-dopaminergic activity and inhibition of hERG channels could impede repositioning of some of these PTH for the treatment of PCa due to potential concerns by regulatory agencies. J54 was designed and tested to be a weaker inhibitor of DR2, and we in fact noticed it had no apparent toxicity in mice, with no extrapyramidal twitches or altered breathing after administration, and relatively weak anti-dopaminergic effects in *C. elegans*. Therefore, it is a bona fide, specific inhibitor of TLK1, and not the DR2, with superior efficacy and better side effects profile.

### Limitations of the Study

Although we tried to ensure mechanistically that J54 works via inhibition of the ADT-activated TLK > Nek1>ATR > Chk1 DDR pathway, we cannot rule out that the tumor regression effects that we have observed could be caused by inhibition of some other target. And in fact, while this paper was under review, a similar PTH with putative lack of DR2 antagonism was described as having antitumor activity via activation of PP2A (Morita et al., 2020). In addition, key large PIKKs, like ATR, ATM, and mTOR are not included in the SCANMAX panel, and therefore could be potentially inhibited by J54. We have shown that ATM can become activated in cells treated with BIC + THD or J54 (Figure S7C), whereas in particular, mTOR has been reported to be inhibited (directly or indirectly) by THD (Kang et al., 2012; Park et al., 2014). Moreover, the combined activation of PP2A and inhibition of mTOR can synergistically suppress growth of pancreatic adenocarcinoma (Allen-Petersen et al., 2019). Another kinase that was inhibited by J54, TTK, may play important roles in mitotic progression and was reported to be critical in pancreatic cancer (Kaistha et al., 2014). Another potential non-kinase target is actually the AR itself (Bisson et al., 2007), which can complicate the interpretation of our experiments, although the fact that mice treated concomitantly with J54 and BIC (which is a much more powerful anti-androgen) show strong tumor regression argues against the idea that the two drugs act on the same pathway.

There are also potential negative consequences in principle to inhibiting TLKs in normal cells and tissues. We have previously reported that in a “normal” non-androgen-dependent cell line (MM3MG) overexpression of TLK1-KD, the cells are viable but a fraction shows defects in mitotic segregation and high levels of aneuploidy (Sunavala-Dossabhoy et al., 2003).

### Resource Availability

#### Lead Contact

Further information and requests for resources and reagents should be directed to and will be fulfilled by the Lead Contact: Arrigo De Benedetti.

#### Materials Availability

Plasmids that were used for this study and derived cell lines are available from the Lead Contact after fulfilling a MTA.

#### Data and Code Availability

All Data and Code generated or analyzed during this study and its Supplemental Information files are available upon request.

## Methods

All methods can be found in the accompanying Transparent Methods supplemental file.

## Supplemental Information

Document S1. Transparent Methods, Figures S1–S8, and Tables S1–S3

Table S4. SCANMAX Table, Related to Figure 1 Kinomescan of J54 inhibitory activity on human kinases.

Data S1. TLK1_Model_PDB File Set, Related to Figure 2 PDB Model of TLK1.

Data S2. Binder Spectral Data, Related to Figure 1 Characterization of J54 and J56.

## Footnotes

Supplemental Information can be found online at https://doi.org/10.1016/j.isci.2020.101474.
