# Supplementary material for: Generation of Phenothiazine with Potent Anti-TLK1 Activity for Prostate Cancer Therapy
Source: iScience. 2020 Aug 20;23(9):101474. doi: 10.1016/j.isci.2020.101474 (PMC7486443; doi:10.1016/j.isci.2020.101474)
Supplement: Document S1. Transparent Methods, Figures S1–S8, and Tables S1–S3 [file mmc1.pdf]

iScience, Volume *23*

Supplemental Information

Generation of Phenothiazine
with Potent Anti-TLK1 Activity
for Prostate Cancer Therapy

Vibha Singh, Siddhant Bhoir, Rupesh V. Chikhale, Javeena Hussain, Donard Dwyer, Richard A. Bryce, Sivapriya Kirubakaran, and Arrigo De Benedetti

TRANSPARENT METHODS:

Molecular Modelling, Docking, Molecular Dynamics Simulations and Free Energy:

Details about methodology used for molecular modelling, docking, molecular dynamics simulations and free energy calculations were explained in details in section A of supplementary material and methods.

Chemical synthesis of J54 and several different Phenothiazines:

Details about chemical synthesis of J54 and several other Phenothiazines, and methodology were explained in section B of supplementary of material and methods.

Protein expression and purification:

The full-length, 65KDa recombinant human TLK1B (hTLK1B) protein for the *in-vitro* experiments was expressed and purified from bacteria using the protocol referenced in main document.

Inhibitor screening, IC_{50} evaluation and mode of binding studies:

The DiscoverRx ADP Hunter™ (Eurofins DiscoverRx, Fremont, CA, US) Kit was used to measure the generation of ADP resulting from kinase phosphorylation of substrate. Details about the inhibitor screening, IC_{50} evaluation and mode of binding studies were explained in section C of supplementary material and methods.

Cell lines:

All prostate cancer cell lines were obtained from the ATCC, recently authenticated, and free from mycoplasma contamination, and cultured as per instructions.

Antibodies:

Antibodies used for present study is listed as supplementary table: Key Resources.

Clonogenic Assay:

One thousand cells were seeded, treated and cultured for over 2 weeks. Colonies were stained with crystal-violet and manually counted by using NIH ImageJ software

Cell proliferation and cell viability Assay:

Ten thousand cells were seeded, and next day treated for desired concentration of J54 and different time-point. Cell proliferation assay was done by crystal-violet staining and viability assay were done by MTT dye.

Cell Cycle Analysis:

We performed cell cycle analysis as described earlier (Singh et al; 2017) with LNCaP and TRAMP-C2 cells with four group of treatment i.e. Control, Bicalutamide, J54 and combination of J54 and Bicalutamide.

Western blot analysis:

Western blot analysis of different proteins listed in supplementary table 1 were done with Control, Bicalutamide, J54 and combination of J54 and Bicalutamide treated samples as described earlier [1] for LNCaP, TRAMP-C2, and VCaP cells.

LNCaP Xenograft Model:

All animals used in this study received humane care based on guidelines set by the American Veterinary, and approved by the Institutional Animal Care and Use Committee (IACUC) of LSU Health Sciences Center at Shreveport and also following ARRIVE (Animal Research: Reporting of In Vivo Experiments) guidelines. Male NOD/SCID mice (5 per group) were purchased from Jackson labs. 10^6 LNCaP cells were injected into the flanks of mice in 0.2 of matrigel. Mice were injected IP bi-weekly with J54: 5 mg/kg body weight (dissolved in DMSO and diluted 1:10 in corn oil). Dose estimation for J54 was derived from pharmacokinetics study. Bicalutamide was dissolved in DMSO and diluted in corn oil 1:10 and administered IP at a dose of 100 mg/kg body weight bi-weekly.

Behavioral assays in *C. elegans* to assess the behavioral effects of J54 as a potential DR2 antagonist (anti-psychotic) were carried out as described in details in Donahoe et al. 2009.

Pharmacokinetic Studies on J54: These were carried out at the preclinical testing lab of UTSW in Dallas, TX. 21 male CD-1 mice were dosed IP with 10 mg/kg J54. 0.2 ml/mouse formulated with 10 % DMSO, 90 % Corn Oil . Whole blood was collected in a synringe coated with ACD. Plasma was processed from whole blood by centrifugation at 10,000 rpm for 10 minutes.

For the standards and QC'S 98 ul & 98.8 ul of blank plasma was added to an eppendorf and spiked with 2 & 1.2 ul of initial standard. Std's, QC's & samples of 100 ul were then crashed with 200ul of methanol containing 0.15% formic acid and 12.5 ng/ml IS (final conc.). The samples were vortexed 15 sec, incubated at room temp for 10' and spun 2x 13,200 rpm in a standard microcentrifuge. The supernatant was then analyzed by LC-MS/MS. Vendor supplied plasma used in standards and QCs: Bioreclamation, LLC; lot #MSE284936; exp = 03/31/2020; ACD anticoagulant. Raw data and additional information can be obtained upon request.

Immunohistochemistry (IHC) on LNCaP Xenograft tumors:

Sectioning and processing of the tissues were carried out in the FWCC Histology Service, using automated processes and equipment to provide uniform and standardized results. Indirect labeling was with ABC Elite: RTU Vectastain Elite Reagent, Vector #PK-7100; DAB: ImmPact DAB, Vector #SK-4105. Light counterstaining was done with hematoxylin.

Statistics:

Significance between treatment groups (mean values and SDs) was performed by a one-way ANOVA followed by Tukey's multiple comparisons tests using GraphPad Prism6 software (GraphPad Software USA). p-value <0.05 (*p < 0.05; **p < 0.01; ***p < 0.001) was considered to indicating a statistically significant difference.

Supplementary Material Methods and Tables

Additional information was included in the SI for the chemical synthesis and physical authentication of the compounds synthesized.

A. Molecular Modelling, Docking, Molecular Dynamics Simulations and Free Energy Calculations:

I. TLK1B

1. TLK1B- Modelling

In the absence of a crystal structure of the TLK1B kinase domain, we constructed a 3D model of its 333 amino acid residues using the ROBETTA protein structure prediction software (Raman et al., 2009) using the hierarchical Ginzu screening method (Song et al., 2013). The modelled TLK1B structure for its stereochemical quality using the MOE software package: the majority of residues were found in the favored and allowed region, with only three residues (Val221, Glu119, Asp233) as outliers (Ramachandran et al., 1963). Good validation scores were obtained for the model using the MolProbity server (Chen et al., 2010). The MolProbity score was 1.52, with a clash score for all atoms to be 0.85 and one outlier rotamer. Further, Qualitative Model Energy analysis (QMEAN) (Benkert et al., 2011) using the QMEAN web server was carried out for the TLK1B model, which gave a QMEAN value of -1.26 which is a good score for modelled proteins, the TLK2 crystal structure (PDB 5O0I) has QMEAN value of -0.85.

1.2. Molecular dynamics simulations of TLK1B protein

MD simulations were performed using the AMBER16 software package (Case et al., 2016). The TLK1B model was immersed in a truncated octahedron of TIP3P water (Mark et al., 2001) giving a total of 17257 water molecules. Na^+ and Cl^- counter ions were added to neutralize the system and provide an ionic strength of 0.1 M. The ff14SB force field (Maier et al., 2015) was used to model the protein.

Simulations were run at 300 K using the Langevin thermostat (Bussi et al., 2008) with a collision frequency of 2 ps^{-1}; and 1 atm using a Monte Carlo barostat (Jorgensen et al., 1996) with volume exchange attempts every 100 fs. A 2 fs integration step was employed. Covalent bonds involving hydrogen were constrained using SHAKE (Debolt et al., 1993). A cutoff of 8 Å was used for short range nonbonded interactions whilst long range electrostatics were treated

using the particle mesh Ewald method (Essmann et al., 1995). Equilibration consisted of rounds of NVT and NPT equilibration for 10 ns in total. Production MD run was performed for 1 μs. Interactions were analysed using the cpptraj (Roe et al., 2013) module, taking configurations every 4 ps. For MD simulations of docked ligand complexes of TLK1B and D2 receptor (see Methods below), each system was immersed in a truncated octahedron of TIP3P water and simulated for 100 ns. Each trajectory was subjected to MM/GBSA analysis (Case et al., 2014; Genheden et al., 2015) for 10,000 equispaced frames.

1.3. Molecular Docking

Molecular docking studies were performed using FRED 3.3.0.3 (McGann et al., 2011 - OpenEye Scientific Software) with the Chemgauss4 score and energy functions (McGann et al., 2003). The TLK1B model was investigated for active sites using SiteFinder tool in MOE software (Paul and Santavy, 2007) The site with highest the PLB score was selected for docking exercise, which was the ATP binding site in the kinase hinge region. The OMEGA module (Hawkins et al., 2010) of OpenEye was used to generate all the possible conformers of the ligands for docking studies. Docking of J54 and THD to the ATP binding site of TLK1B was performed. For docking to the D2 receptor, the crystal structure of D2 receptor bound to risperidone was obtained from the Protein Data Bank (PDB 6CM4) (Wang et al., 2018). The crystal structure was analysed for any inconsistencies like breaks in the sequence and protonation states using the MOE software. The protein was further prepared by energy minimisation and addition of hydrogens to reduce the strain energy from crystal formation. Conformers of ligands J54, THD and risperidone were docked using FRED.

B. Chemical synthesis of J54 and several different Phenothiazine:

Chemical reagents and Instrumentation:

All the solvents were of HPLC grade and procured from Sigma, Finar and SD Fine. And all other reagents were of analytical grade and were used without further purification. The reaction was performed in a round-bottomed flask with magnetic stirring bar under N_2 atmosphere. Completion of the reaction was monitored by the Thin-layer chromatography carried on Merck TLC silica gel 60 F254 and visualized by ultraviolet irradiation. The purification of all the synthesized compounds was done by the column chromatography using silica gel 100-200 mesh supplied by Merck. The synthesized compounds were characterized by NMR spectra,

recorded by Bruker AVANCE III 500 (^{1}H NMR: 500 MHz, ^{13}C NMR: 125 MHz). The splitting patterns are referred as follows: s, singlet; d, doublet; t, triplet; m, multiplet; dd, doublet of doublets; and td, triplet of doublets. NMR spectra were comparable with those previously published (J3-56). Melting points (mp) were recorded in an open glass capillary using LABINDIA Visual Melting Range Apparatus (MR-Vis) instrument. The mass spectrum and the infrared spectra (wave numbers, cm^{-1}) were recorded by Waters Synapt-G2S ESI-Q-TOF (Positive mode) and Perkin Elmer FTIR Spectrometer (Spectrum 2) respectively.

S1 : Synthesis of 4-(2-(10H-phenothiazin-10-yl) ethyl) morpholine, J-54

The compound **J-54** was initially tried to synthesized in 1949 (Dahlabom et al., 1949) but it was not isolated or purified in a pure state. We are the first one to report **J3-54**, crystalline solid, which is in pure state.

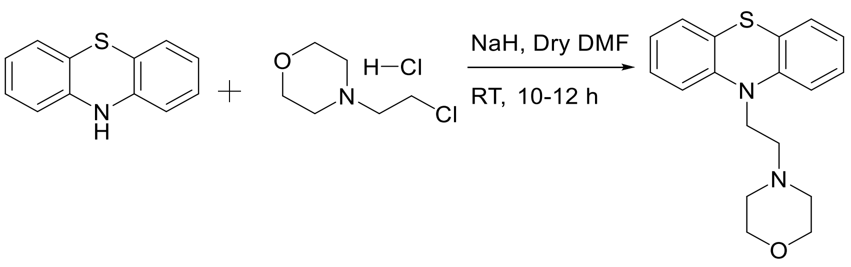

J - 54 (59 % yield)

Synthetic procedure

To a solution of phenothiazine (200 mg, 1.0 mmol) in dry DMF in a round-bottomed flask, sodium hydride (96.4 mg, 4 mmol) was added slowly under inert condition at 0 – 5 °C. The reaction mixture was stirred at room temperature for 15 – 20 min followed by the addition of 4-(2-chloroethyl) morpholine hydrochloride (186 mg, 1 mmol). The above reaction mixture was stirred at room temperature for 10 – 12 h. The reaction towards completion was monitored by TLC. Then the solution was diluted with ethyl acetate and washed with brine solution. The organic layer was dried over Na_2SO_4, evaporated on a rotary evaporator under high pressure. The crude product was purified by silica gel column chromatography using 4 % ethyl acetate/Hexane, yielding the desired product as a yellowish orange solid. Yield (185 mg, ~ 59 %); mp: 78 – 80 ºC; IR (ν, cm^{-1}): 1117(C– O), 1035 (C–N); ^{1}H NMR (500 MHz, $CDCl_3$): δ 7.11 (m, 4H), 6.9 (m, 4H), 4.0 (t, 2H, J = 7 Hz), 3.71 (t, 4H, J = 4.5 Hz), 2.72 (t, 2H, J = 7 Hz), 2.51 (t, 4H, J = 4.5 Hz); ^{13}C NMR (125 MHz, $CDCl_3$): δ 145.0, 127.5, 127.2, 124.9, 122.6, 115.4, 66.9, 56.0, 54.0, 45.7; HRMS (*m/z*): $[M+H]^+$ calculated for $C_{18}H_{21}N_2OS$, 313.1330; found, 313.1336.

S2: Synthesis of 10-methyl-10H-phenothiazine, J-56

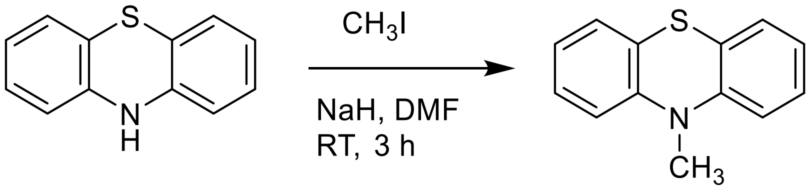

J-56, 88% yield

The procedure was analogous to that used for the synthesis of **J-54** except that methyl iodide was used instead of 4-(2-chloroethyl) morpholine hydrochloride. The crude product was purified by silica gel column chromatography using 0.1 % ethyl acetate/Hexane, yielding the desired product as a white solid.[22] Yield(189 mg, ~ 88 %); mp: 100 – 102 °C; IR (ν, cm^{-1}): 1330(C–N); ^{1}H NMR (500 MHz, $CDCl_3$): δ 7.12 (m, 2H), 6.91 (t, 2H, J = 6.5 Hz), 6,79 (d, 2H, J = 8.5 Hz), 3.3 (s, 3H); ^{13}C NMR (125 MHz, $CDCl_3$): δ 145.8, 127.4, 127.1, 123.4, 122.4, 114,0, 35.3; HRMS (*m/z*): $[M+H]^+$ calculated for $C_{13}H_{12}NS$, 214.0690; found, 214.0714.

Acetylation(**J-55**), benzylation(**J-50**) (Burger et al., 1954), Methylation(**J-56**) (Das et al., 2009) and benzoylation(**J-66**) (Darvesh et al., 2007) of the phenothiazine molecule were performed by the previously reported procedure (with considerable modification) and named as follows:

S3: General Synthetic Scheme for the compounds having same parent scaffold, phenothiazine

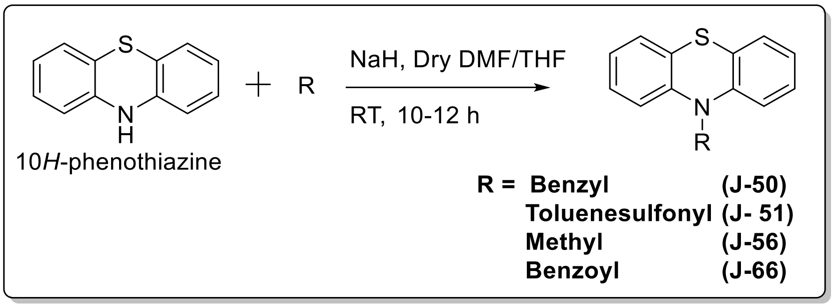

Supplementary Table 1. List of the chemical structure of **J-50**, **J-51**, **J-55**, **J-65** and **J-66** compounds:

S.I.	Chemical structure with batch number	S.I.	Chemical structure with batch number
1	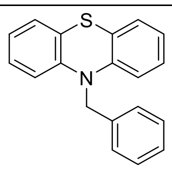 **J-50**	5	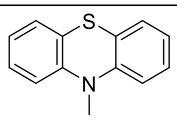 **J-56**
2	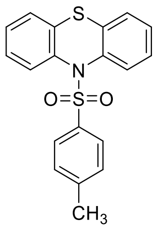 **J-51**	6	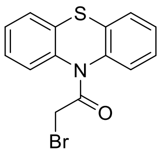 **J-65**
3	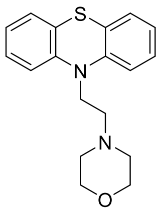 **J-54**	7	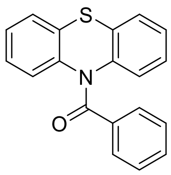 **J-66**
4	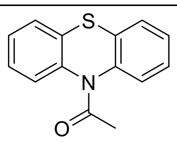 **J-55**		

C. Inhibitor screening, IC_{50} evaluation and mode of binding studies:

I. Inhibitor screening, IC_{50} evaluation and mode of binding studies:

The DiscoverRx ADP Hunter™ (Eurofins DiscoverRx, Fremont, CA, US) assay was performed to measure the generation of ADP resulting from kinase phosphorylation of substrate. The enzyme-coupled reaction produces a positive fluorescent signal that is directly proportional to the ADP in the solution and hence, the activity of the kinase. The assay was carried out in triplicates in a solid-black, 96-well plate in a volume of 40 µl solvent containing

protein kinase (e.g. hTLK1B in this case), kinase substrate (e.g. Nek1 peptide in this case), ATP and 1X Kinase Reaction Buffer (KRB). The 1X KRB comprised of 15 mM Hepes, 20 mM NaCl, 1 mM EGTA, 0.02 % Tween 20, 10 mM MgCl2, and 0.1 % bovine gamma globulins, at pH 7.4. Reactions in each well were started immediately by adding ATP and kept going for half an hour at room temperature. 40 µl of proprietary Reagent A followed by 20 µl of proprietary Reagent B were then added to each well to deplete the unconsumed ATP, terminate the kinase reaction and convert ADP to ATP. In the end, 10 µl of a stop solution was added to each well to allow the newly synthesised ATP to be measured using a detectable resorufin fluorescence signal. The amounts of kinase, substrate, ATP, reaction temperature and incubation times were optimised for the optimal performance of the assay. For the inhibitor screening experiments, 2 µl of Staurosporine, THD, and the test compounds (J3-50, J3-51, J3-54, J3-55, J3-56, J3-57, J3-59, J3-60, J3-65, and J3-66) were used at a final concentration of 20 µM in the reaction. DMSO was used as a suitable control vehicle. For the dose-response curves and the mode of binding studies, the control and the test inhibitors, and the ATP concentrations were varied to get a twelve-point data set. The fluorescence signal generated was measured using a Tecan Infinite® M1000 Pro Plate Reader (Tecan Group Ltd., Mannedorf, Switzerland) using excitation/emission wavelengths of 530/590 nm with an integration time of 0.1 s. Statistical analyses were performed using GraphPad Prism 6 statistical software program (GraphPad Software Inc., CA, USA), and the fitting of the kinetic and dose-response data was done globally using a multiresponse non-linear regression model to get a best-fit value that applies to all data sets. Suitable negative control wells without the protein kinase, substrate and ATP were also included in the kinase assay.

D. Quantitation of human TLK1 transcript variant 3 (qRT-PCR):

We performed RNA isolation and RT-PCR analysis as described earlier (Singh et al., 2019) with LNCaP cells. TLK1 (NM_12290) primers from IDT (PrimeTime qPCR Primer Assays) were used and transcript levels were normalized by GAPDH levels..

TLK1B - Primer 1: GCAACTCCAGTAAATCTAGCTTCC.
TLK1B - Primer 2: GTCCCTGCTGAATCACACG.]

E. Behavioral assays in *C. elegans* to assess the behavioral effects of J54 as a potential DR2 antagonist (anti-psychotic) were carried out as described in details in (Donohoe et al., 2006; Donohoe et al., 2009).

<u>F. Pharmacokinetic Studies on J54:</u> These were carried out at the preclinical testing lab of UTSW in Dallas, TX. 21 male CD-1 mice were dosed IP with 10 mg/kg J54. 0.2 ml/mouse formulated with 10 % DMSO, 90 % Corn Oil . Whole blood was collected in a synringe coated with ACD. Plasma was processed from whole blood by centrifugation at 10,000 rpm for 10 minutes. For the standards and QC'S 98 ul & 98.8 ul of blank plasma was added to an eppendorf and spiked with 2 & 1.2 ul of initial standard. Std's, QC's & samples of 100 ul were then crashed with 200ul of methanol containing 0.15% formic acid and 12.5 ng/ml IS (final conc.). The samples were vortexed 15 sec, incubated at room temp for 10' and spun 2x 13,200 rpm in a standard microcentrifuge. The supernatant was then analyzed by LC-MS/MS. Vendor supplied plasma used in standards and QCs: Bioreclamation, LLC; lot #MSE284936; exp = 03/31/2020; ACD anticoagulant. Raw data and additional information can be obtained upon request.

Supplementary Table 2 – related to Fig. 4

Cell line	Sub-G1	G1	S	G2/M
**LNCaP** Control	0%	93%	4%	3%
+BIC	2%	94%	2%	2%
+J54	1%	94%	3%	2%
+BIC+J54	33%	53%	4%	10%
**TRAMP-C2** Control	3%	42%	26%	29%
+ BIC	7%	58%	21%	14%
+J54	11%	46%	29%	14%
+BIC+J54	26%	27%	21%	26%

Sup Table 3 Inhibitory Effect of Test Substances on Radioligand Binding to two recombinant Human Dopamine Receptors (DR2)

Assay system	Inhibition ratio (%)			
	J54	THD	Positive substance	
	1×10^{-7} mol/L	1×10^{-7} mol/L	1×10^{-5} mol/L	
Dopamine D1 (Human)	12.17	67.69	100.00	*R*(+)-SCH-23390
Dopamine D3 (Human)	19.77	95.57	99.95	(±)-7-OH-DPAT

Receptor: Human recombinant, PKI, Cat No. 6110513

50 mmol/L Tris-HCl (pH 7.4) containing 120 mmol/L NaCl, 5 mmol/L KCl, 2 mmol/L $MgCl_2$ and 1 mmol/L $CaCl_2$

Tracer: SCH23390, [N-methyl-^{3}H]-, PKI, Cat No. NET930

Receptor: Human recombinant, PKI, Cat No. ES-173-M 50 mmol/L Tris-HCl (pH 7.4) containing 5 mmol/L $MgCl_2$

Tracer: 7-Hydroxy DPAT, R-(+)-[^{3}H]-, PKI, Cat No. NET1169

Duplicate samples were analyzed and averaged results are shown.

Data Processing

Inhibition ratios (%) were calculated from “100 – binding ratio”.

Binding ratio: $[(B–N)/(B_0–N)]\times100$ (%)

B: Bound radioactivity in the presence of the test article (individual value)

B_0: Total bound radioactivity in the absence of the test article (mean value)

N: Non-specific bound radioactivity (mean value)

Key Resources:

S. No.	Antibody Name	Catalog number	Company
**1**	TLK1 antibody [N2C2]	GTX102891	Genetex
**2**	Rabbit NEK1 Antibody	A304–570A	Bethyl Lab
**3**	Purified custom pNek1 antibody	custom	Thermofisher
**4**	H2A.X (Ser139)	05-636	Millipore
**5**	Cleaved Caspase-3	9579	Cell signaling technology
**6**	Cleaved Caspase-3	9661	Cell signaling technology
**6**	GAPDH	2118S	Cell signaling technology
**7**	Cleaved PARP (Asp214)(D64E10	5625	Cell signaling technology
**8**	Anti-PCNA Antibody	MAB424	Millipore
**9**	Phospho-Chk1 (Ser317)	2344	Cell signaling technology
**10**	Phospho-ATR (Thr1989)	5801S	Cell signaling technology
**11**	Alpha mouse HRP	7076	Cell signaling technology
**12**	Ki-67 (D3B5)	12202	Cell signaling technology
**13**	p21 Waf1/Cip1	2946	Cell signaling technology
**14**	Alpha rabbit IgG-HRP	7074S	Cell signaling technology
**15**	Anti-beta Tubulin antibody Loading Control (HRP)	Ab218	Abcam

References

Benkert, P., Biasini, M., Schwede, T. (2011). Toward the estimation of the absolute quality of individual protein structure models. Bioinformatics 23, 343-50.

Burger, A. (1954). Some derivatives of phenothiazine. J. Org. Chem 19, 1113-1116.

Bussi, G., Parrinello, M. (2008). Stochastic thermostats: comparison of local and global schemes. Comput. Phys. Commun. 179, 26-29.

Case D. A., Betz, R. M., Kollman, P.A. (2016). Amber 16. Univ. California, San Fr.

Case D. A., Betz, R. M., Madej, K.M, V.B. (2014). The Amber Molecular Dynamics Package. AMBER 14. Univ. California, San Fr.

Chen, V.B., Arendall, W.B., Headd, J.J., Keedy, D.A., Immormino, R.M., Kapral, G.J., Murray, L.W., Richardson, J.S., Richardson, D.C. (2010). MolProbity: All-atom structure validation for macromolecular crystallography. Acta Crystallogr. Sect. D Biol. Crystallogr 66, 12-21.

Dahlbom, R. (1949). Antihistamine agents. IV. Piperidino and morpholinoalkyl derivatives of phaenothiazine. Act Chem Scand 2, 247-255.

Debolt, S.E., Kollman, P.A. (1993). AMBERCUBE MD, parallelization of Amber's molecular dynamics module for distributed-memory hypercube computers. J. Comput. Chem 14, 312-329.

Darvesh, S., McDonald, R. S., Darvesh, K. V., Mataija, D., Conrad, S., Gomez, G., Walsh, R., Martin, E. (2007). Selective reversible inhibition of human butyrylcholinesterase by aryl amide derivatives of phenothiazine. Bioorganic Med. Chem 15, 6367–6378.

Das, B., Ravikanth, B., Kumar, A. S., Kanth, B. S. (2009). An efficient procedure for the synthesis of substituted pyridines using kf·Al2O3. J. Heterocycl. Chem 46, 1208–1212.

Donohoe, D.R., Aamodt, E.J., Osborn, E., and Dwyer, D.S. (2006) Antipsychotic drugs disrupt normal development in Caenorhabditis elegans via additional mechanisms besides dopamine and serotonin receptors. *Pharmacological research* 54:361-372.

Donohoe, D.R., Jarvis, R.A., Weeks, K., Aamodt, E.J., and Dwyer, D.S. (2009) Behavioral adaptation in C. elegans produced by antipsychotic drugs requires serotonin and is

associated with calcium signaling and calcineurin inhibition. *Neuroscience research* 64:280-289.

Essmann, U., Perera, L., Berkowitz, M.L., Darden, T., Lee, H., Pedersen, L.G. (1995). A smooth particle mesh Ewald method. J. Chem. Phys 103, 8577-8593.

Genheden, S., Ryde, U. (2015). The MM/PBSA and MM/GBSA methods to estimate ligand-binding affinities. Expert Opin. Drug Discov 10, 449-461.

Hawkins, P.C.D., Skillman, A.G., Warren, G.L., Ellingson, B.A., Stahl, M.T. (2010). Conformer generation with OMEGA: Algorithm and validation using high quality structures from the protein databank and cambridge structural database. J. Chem. Inf 50, 572-584.

Jorgensen, W.L., Tirado-Rives, J. (1996). Monte Carlo vs molecular dynamics for conformational sampling. J. Phys. Chem 100, 14508-14513.

Maier, J.A., Martinez, C., Kasavajhala, K., Wickstrom, L., Hauser, K.E., Simmerling, C. (2015). ff14SB: Improving the Accuracy of Protein Side Chain and Backbone Parameters from ff99SB. J. Chem. Theory Comput 11, 3696-3713.

Mark, P., Nilsson, L. (2001). Structure and dynamics of the TIP3P, SPC, and SPC/E water models at 298 K. J. Phys. Chem. A 105, 9954-9960.

McGann, M. (2011). FRED pose prediction and virtual screening accuracy. J. Chem. Inf. Model 51, 578-596.

McGann, M.R., Almond, H.R., Nicholls, A., Grant, J.A., Brown, F.K. (2003). Gaussian docking functions. Biopolymers 68, 76-90.

Paul Labute and Martin Santavy (2007). Locating Binding Sites in Protein Structures [WWW Document]. (J. Chem. Comput. Gr).

Ramachandran, G.N., Ramakrishnan, C., Sasisekharan, V. (1963). Stereochemistry of polypeptide chain configurations. J. Mol. Biol 7, 95-99.

Raman, S., Vernon, R., Thompson, J., Tyka, M., Sadreyev, R., Pei, J., Kim, D., Kellogg, E., Dimaio, F., Lange, O., Kinch, L., Sheffler, W., Kim, B.H., Das, R., Grishin, N. V., Baker, D. (2009). Structure prediction for CASP8 with all-atom refinement using Rosetta. Proteins Struct. Funct. Bioinforma 77, 89-99.

Roe, D.R., Cheatham, T.E. (2013). PTRAJ and CPPTRAJ: Software for processing and analysis of molecular dynamics trajectory data. J. Chem. Theory Comput 9, 3084-3095.

Singh, V., Jaiswal, P., Ghosh, I., Koul, H. K., Yu, X., De Benedetti, A. (2019). Targeting the TLK1/NEK1 DDR axis with Thioridazine suppresses outgrowth of Androgen Independent Prostate tumors. International journal of cancer 145, 1055-1067.

Song, Y., Dimaio, F., Wang, R.Y.R., Kim, D., Miles, C., Brunette, T., Thompson, J., Baker, D. (2013). High-resolution comparative modeling with RosettaCM. Structure 21, 1735-1742.

Wang, S., Che, T., Levit, A., Shoichet, B.K., Wacker, D., Roth, B.L. (2018). Structure of the D2 dopamine receptor bound to the atypical antipsychotic drug risperidone. Nature 555, 269-273.

Fig S1. **Overlay of J54 and THD ligands within the active site of TLK1 – related to Fig. 2**, from various viewpoints.

Fig S2. Docked pose of ligands to active site of D2 dopamine receptor– related to Fig. 2D, after 100 ns of MD simulation: (a) J54, (b) THD and (c) risperidone.

Fig. S3 H^3NMR and MS of J54 – related to Fig. 1, "inhibitory compounds" figure.

Fig. S4 H^3NMR and MS of J3-56 - **related to Fig. 1**, "inhibitory compounds" figure.

Fig. S5 Pharmacokinetics plasma distribution of J54 – related to Fig. 6. These were carried out by Noelle Williams, Director of Preclinical Pharmacology Core at UTSW, and additional information on the study and data/analyses is available upon request.

Fig. S6 Behavioral assays in *C. elegans* - Related to Fig. 2D – reduced of DR2 binding activity. **A** For the **pharyngeal pumping** assay, wild-type animals (N2 strain) were transferred to plates with diluted DMSO (Control) or drug (J-54 or trifluoperazine) at 160 μM final concentration for 90 min before quantifying pharyngeal pumping see SI methods). We counted visible movement of the grinder (pharyngeal contractions) for 30 sec to obtain the pumping rate (N = 45). Note that in pharyngeal pumping, J54 reduced the pumping rate but to a lesser extent than a much lower concentration of TFP. Inhibition of pharyngeal pumping by trifluoperazine (TFP), a typical PTH antipsychotic, is attributed to its known activity as a dopamine and calmodulin antagonist (40). **B**. **Foraging**, regulated by serotonin and dopamine, was studied in wild-type animals evaluated in the presence of DMSO (Control) or J54 at a high dose of 160 µM vs. TFP at 40 µM. The number of omega turns (head touches body) and reversals were then counted over the next 3 min to quantify search behavior for each group (N = 13 per group). **C**. **Reduction of dopamine-induced immobility**. Dopamine produces immobility in *C. elegans* after 2-3 hr of exposure. Antipsychotic drugs, such as haloperidol, that potently block D2 dopamine receptors largely counteract the effects of dopamine, i.e., more animals continue to

move on plates that include dopamine plus drug. Wild-type animals were incubated on 60 agar plates with bacteria and diluted DMSO (Control) or J54 or haloperidol at 160 μM final concentrations. After 1 hr on these plates, they were transferred to 60 mm agar plates with bacteria that also contained dopamine (final concentration 25 mM) plus diluted DMSO (Control) or else J-54 or haloperidol at final concentrations of 160 μM. After 3 hr on the dopamine plates, we examined movement and counted animals as moving if they traversed half their body length in either the forward or backward direction during a 5-sec observation period. We then calculated the percentage of animals moving and repeated this experiment three times to confirm the effects of drug. J54 was not toxic to worms even after prolonged exposure.

Fig. S7 A. H&E stain of representative LNCaP tumors (from 3 mice per group) - Related to Fig. 6. Note that in the BIC+J54 tumor, there is lesser cancer cellularity, with "empty" spaces interspersed with fibrous tissue, clearly suggesting the combination is having an effect in reducing tumor growth or killing it. **B) qRT-PCR quantitation of TLK1B mRNA expression** (from 3 LNCaP samples per group in triplicate reactions). **C) Activation of pATM-S1981 following 1 day incubation of LNCaP and TRAMP-C2 cells with BIC and J54 D) Reduction of Rad9 phosphorylation by J54 and THD – related to Fig. 5.** LNCaP cells were treated for 12h with 10 μM J54 or THD, and pRad9-S328 or total Rad9 were determined by WB.

Fig. S8. IHC analysis of markers of proliferation (Ki67 - A), apoptosis (Cleaved PARP and Caspase 3 – B, C), and presence of γH2AX (an indicator of DSBs – related to Fig. 6. Quantitation of the stained sections is shown on the side. Representative section from 3 mice per group are shown. Two way-Anova tests were done to compare the groups for statistical significance. Related to Fig. 6.

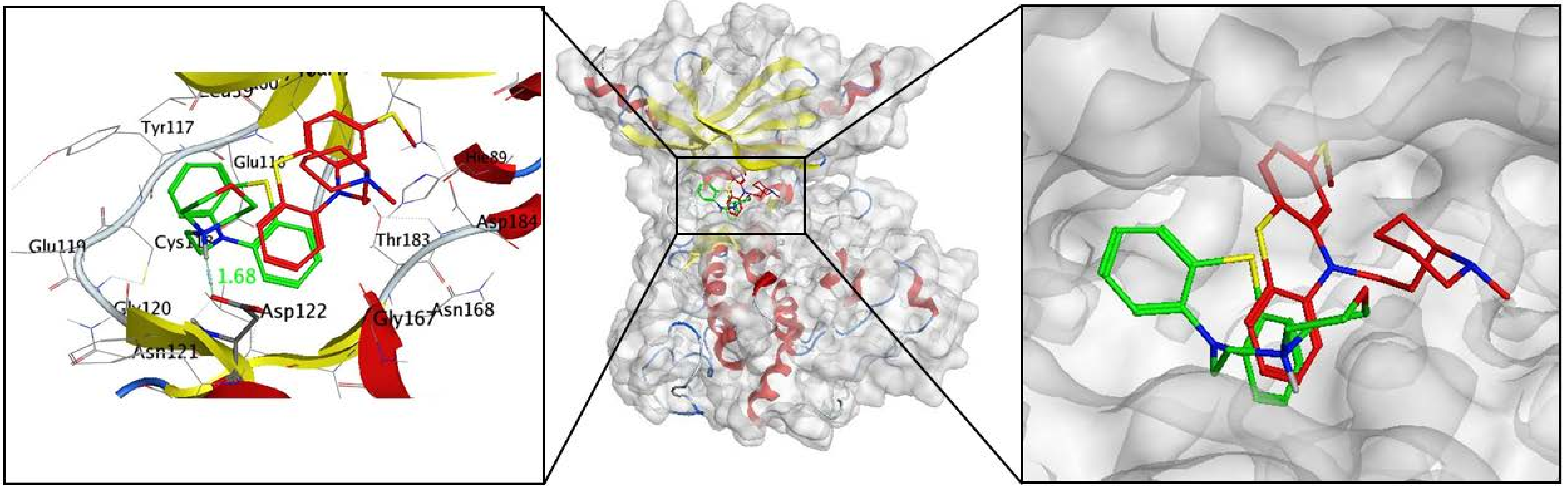

Tyr117
Thr183
Asp184
1.68
Gly120
Asp122
Gly167
Asn168
Asn121

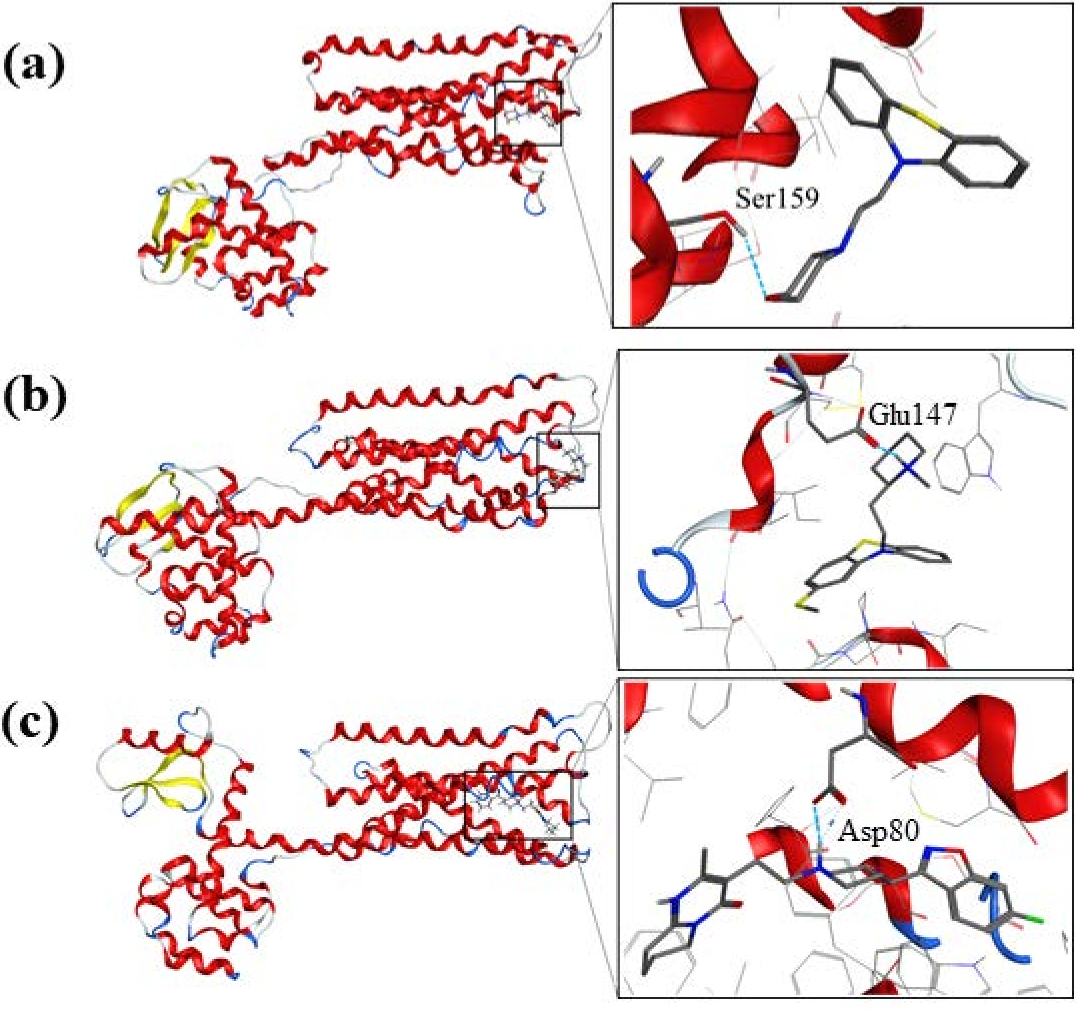
(a)
Ser159
(b)
Glu147
(c)
Asp80

A.

^{1}HNMR of J3-54

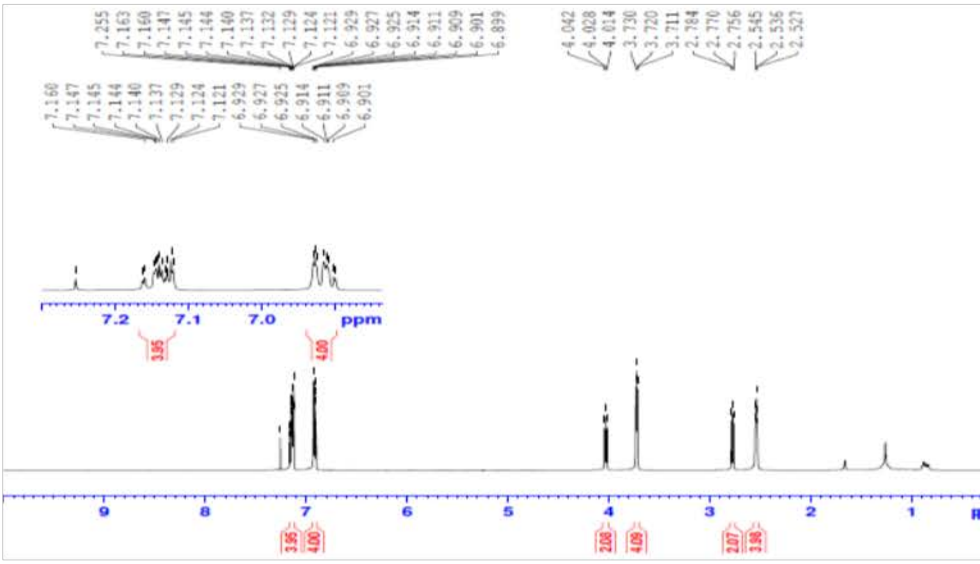

B.

^{13}CNMR of J3-54

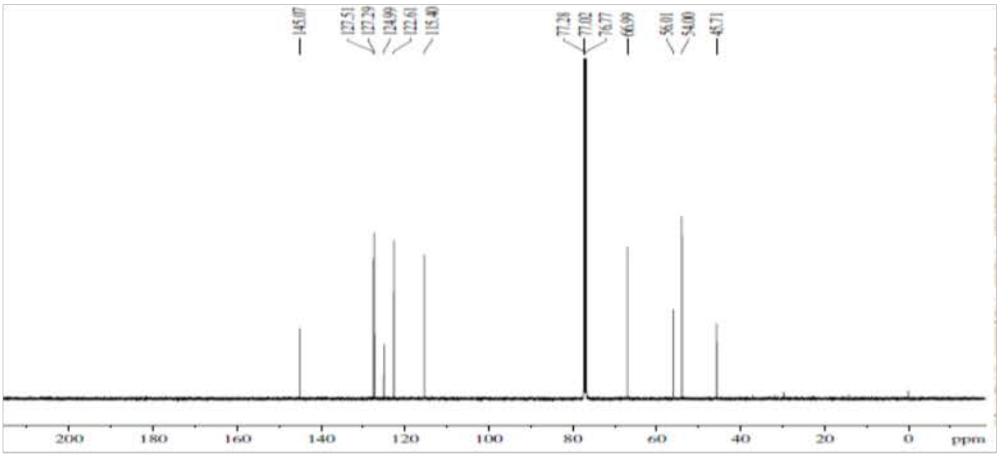

C.

Mass Spectra of J3-54

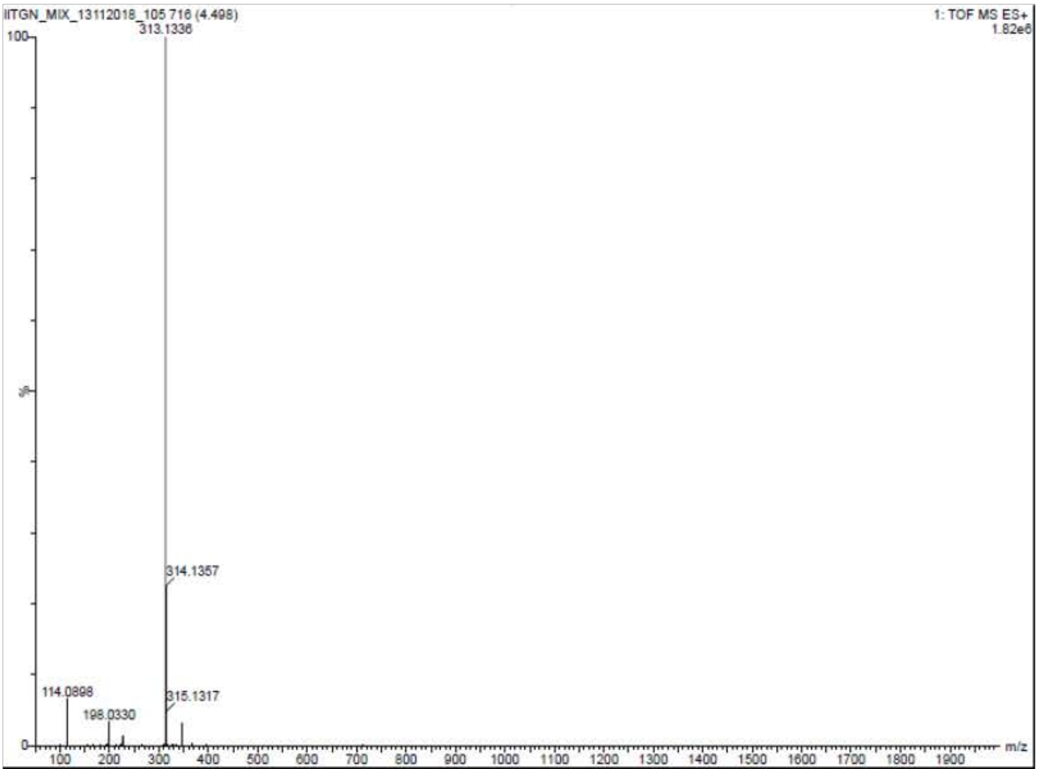

A.

^{1}HNMR of J3-56

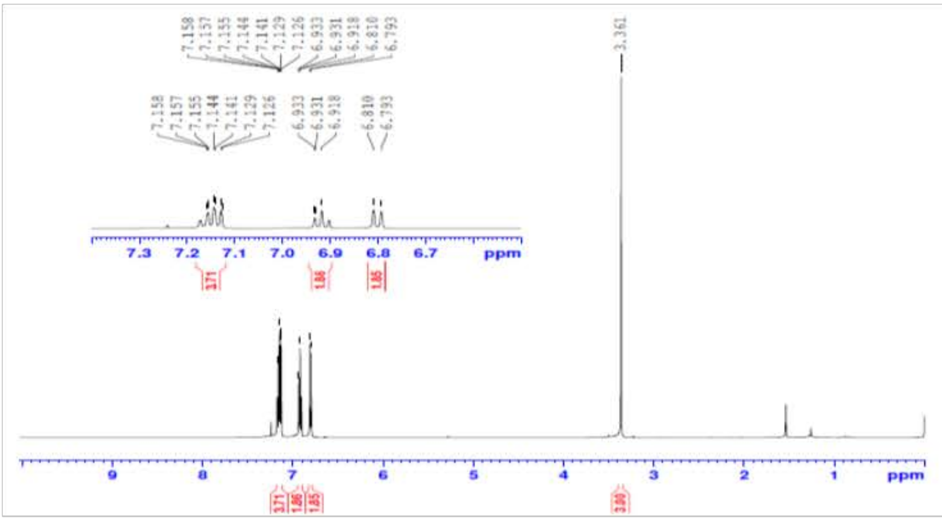

B.

^{13}CNMR of J3-56

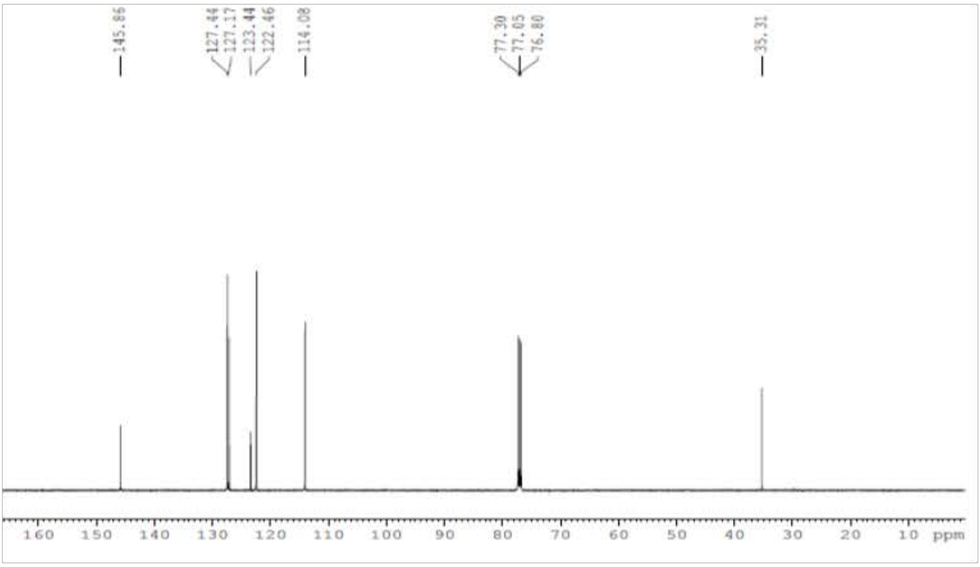

C.

Mass spectra of J3-56

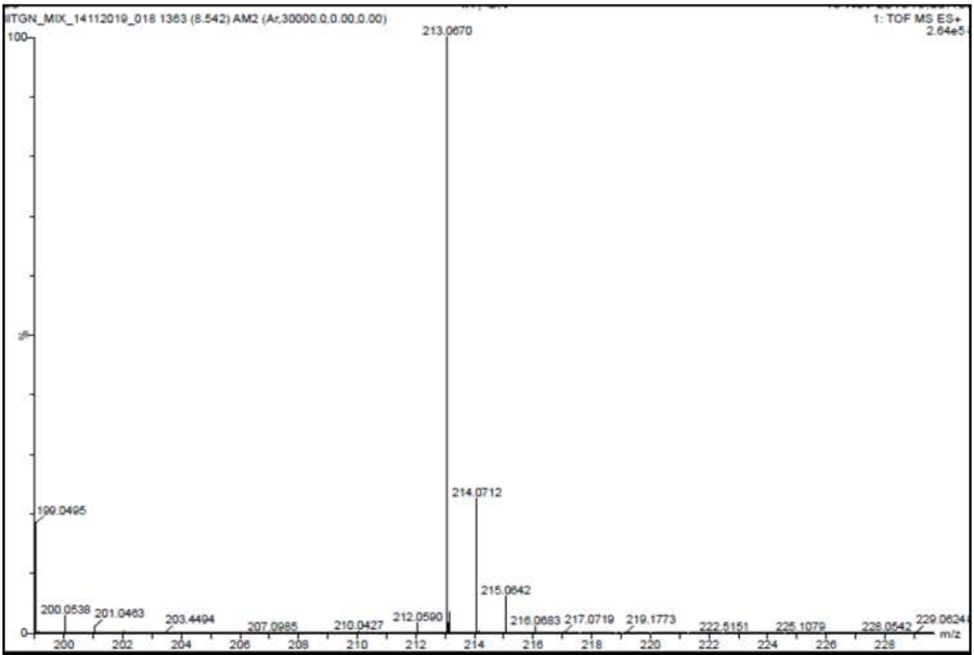

A.

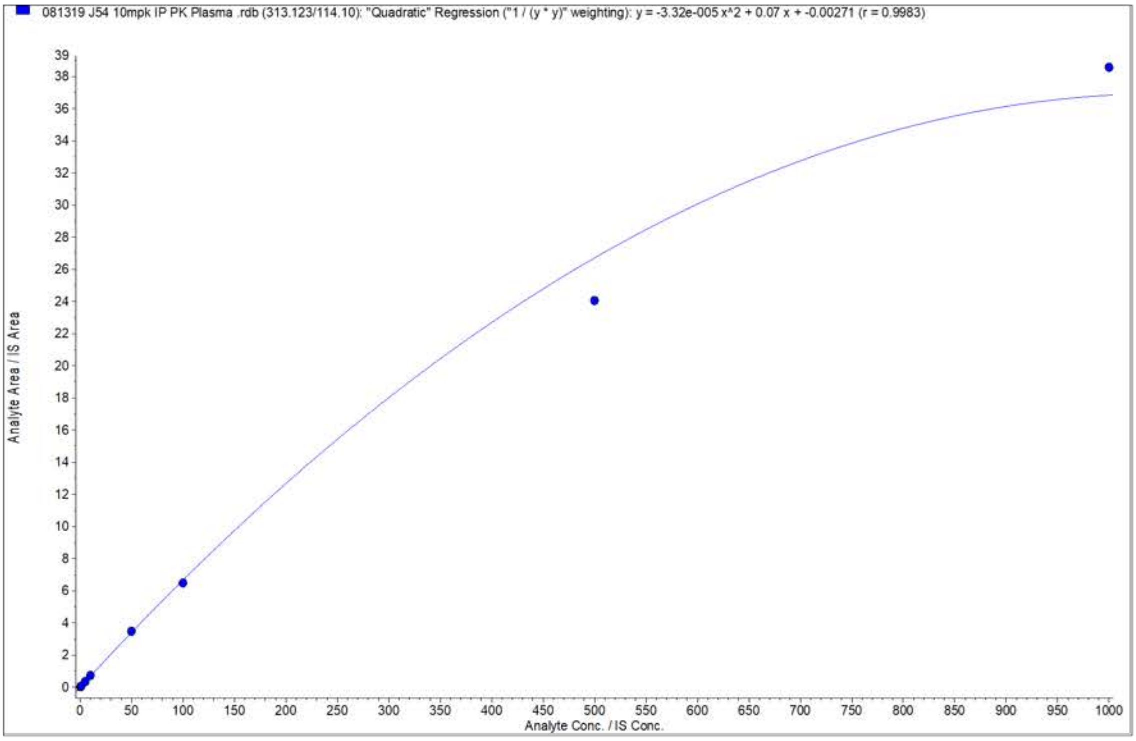
081319 J54 10mpk IP PK Plasma .rdb (313.123/114.10): "Quadratic" Regression ("1 / (y * y)" weighting): y = -3.32e-005 x^2 + 0.07 x + -0.00271 (r = 0.9983)
Analyte Area / IS Area
Analyte Conc. / IS Conc.

B.

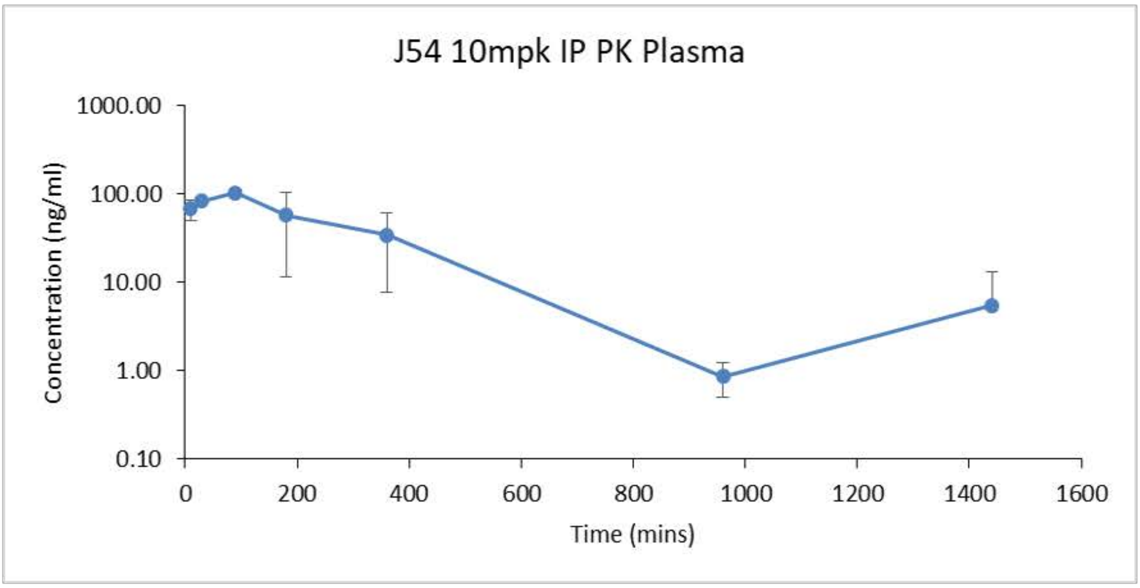
J54 10mpk IP PK Plasma
Concentration (ng/ml)
Time (mins)

C.

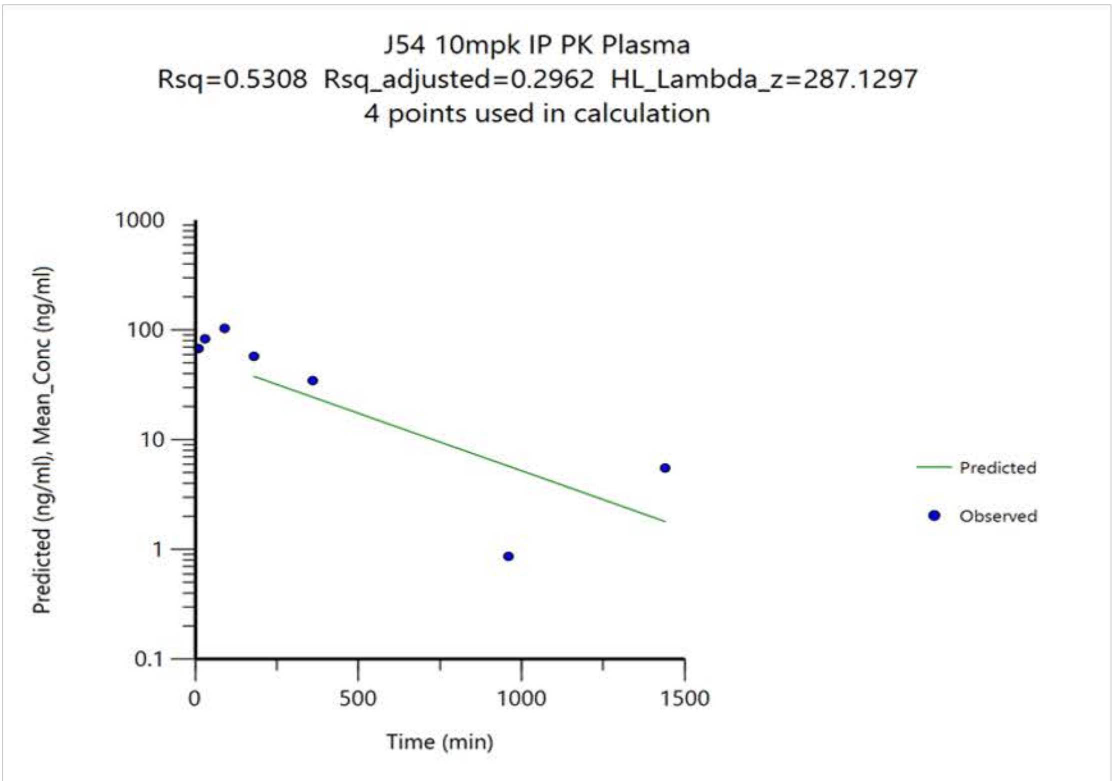
J54 10mpk IP PK Plasma
Rsq=0.5308 Rsq_adjusted=0.2962 HL_Lambda_z=287.1297
4 points used in calculation
Predicted (ng/ml), Mean_Conc (ng/ml)
Time (min)
Predicted
Observed

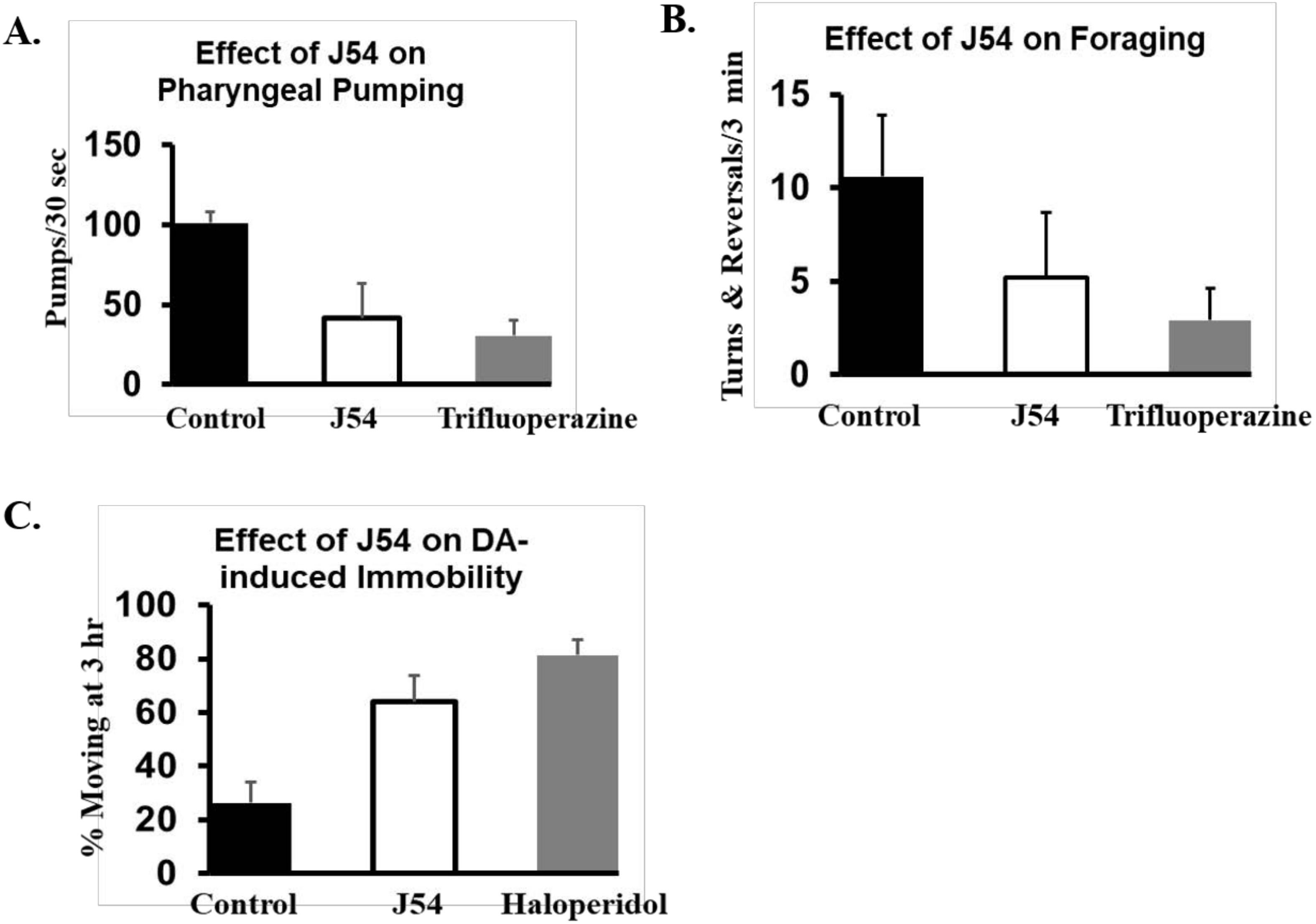
A.
Effect of J54 on Pharyngeal Pumping
Pumps/30 sec
150
100
50
0
Control
J54
Trifluoperazine
B.
Effect of J54 on Foraging
Turns & Reversals/3 min
15
10
5
0
Control
J54
Trifluoperazine
C.
Effect of J54 on DA-induced Immobility
% Moving at 3 hr
100
80
60
40
20
0
Control
J54
Haloperidol

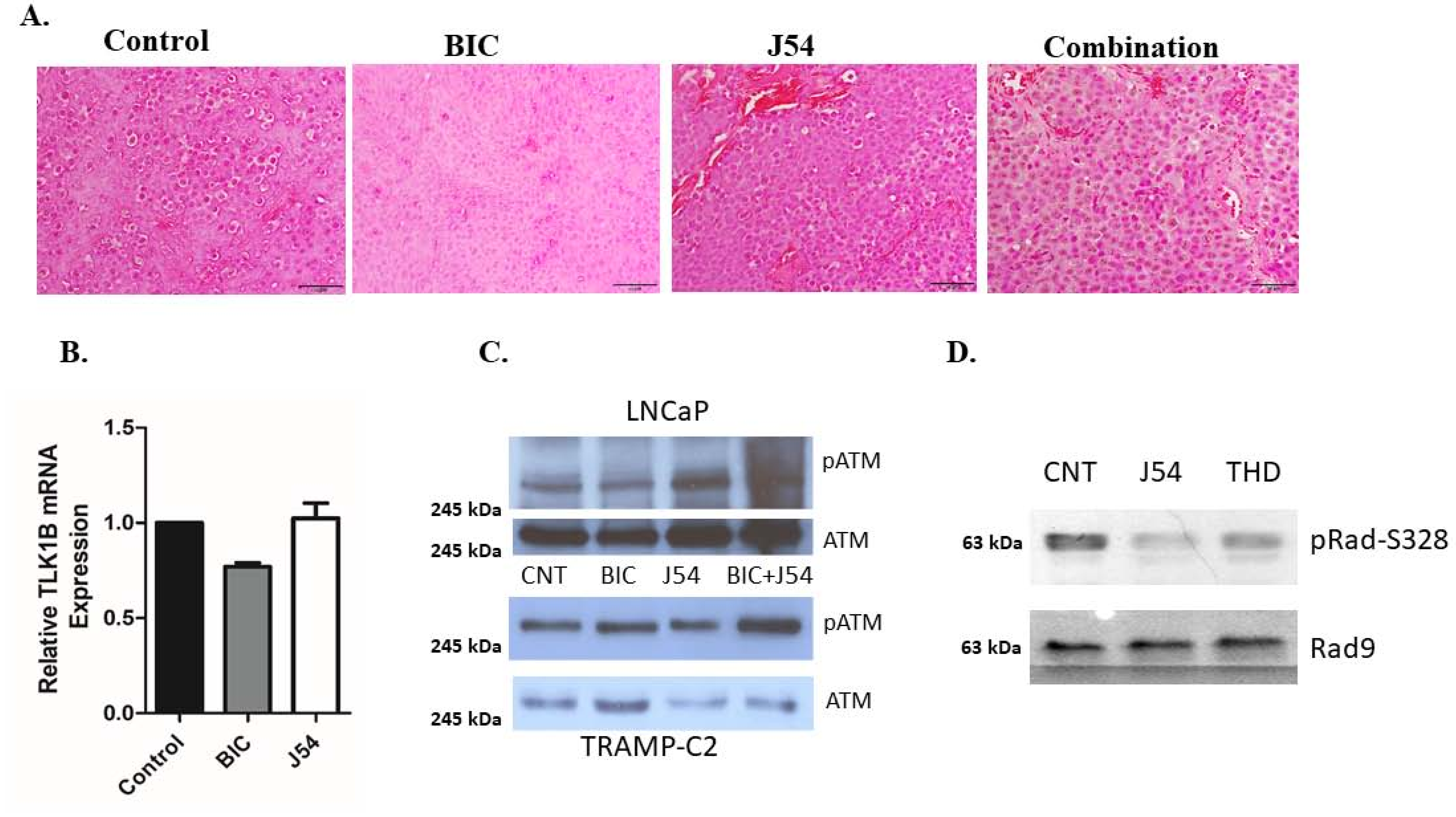
A.
Control
BIC
J54
Combination
B.
Relative TLK1B mRNA Expression
1.5
1.0
0.5
0.0
Control
BIC
J54
C.
LNCaP
pATM
ATM
245 kDa
245 kDa
CNT
BIC
J54
BIC+J54
pATM
ATM
245 kDa
245 kDa
TRAMP-C2
D.
CNT
J54
THD
63 kDa
pRad-S328
63 kDa
Rad9

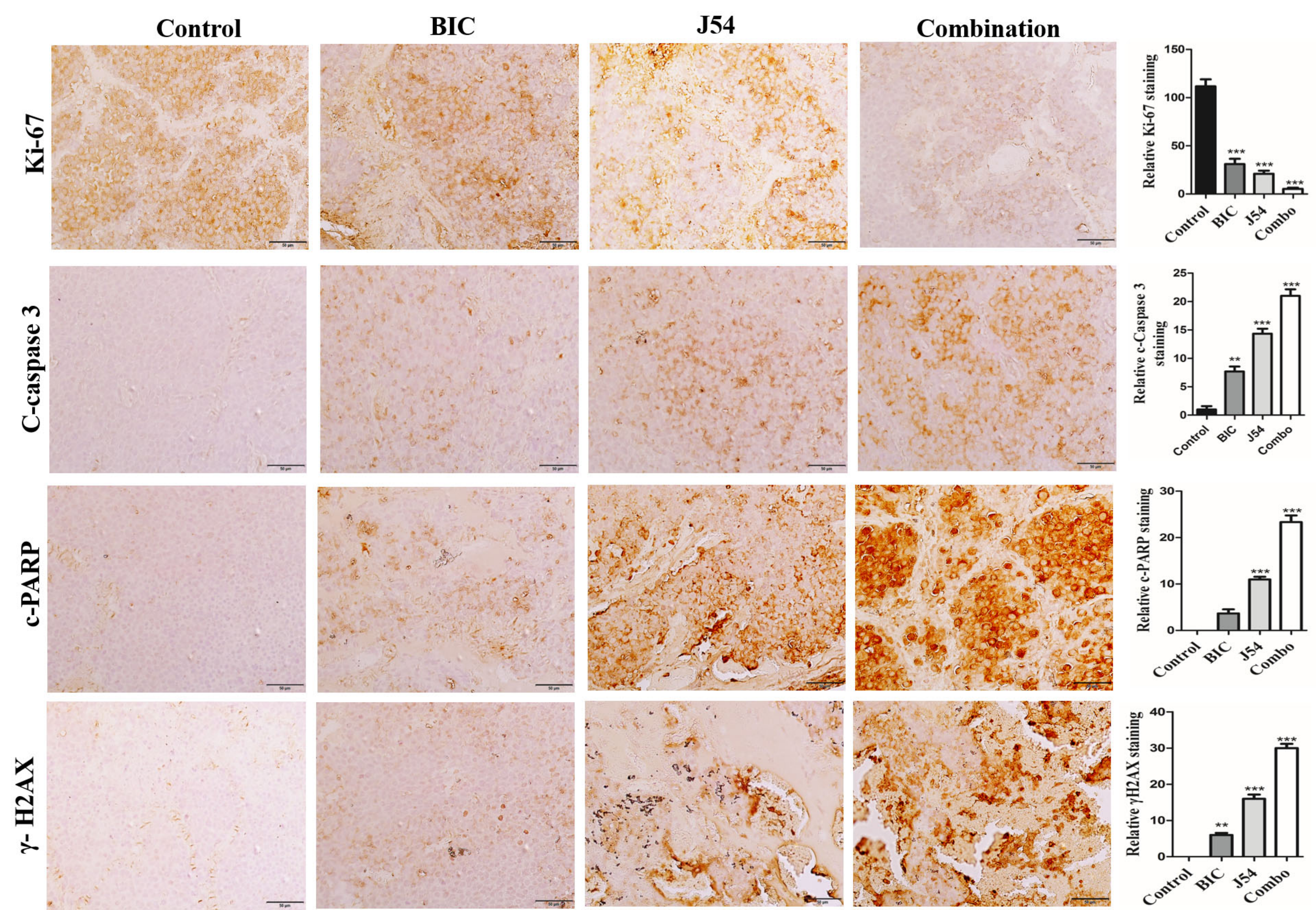

Control
BIC
J54
Combination
Ki-67
C-caspase 3
c-PARP
γ- H2AX
Relative Ki-67 staining
150
100
50
0
***
***
***
Control
BIC
J54
Combo
Relative c-Caspase 3 staining
25
20
15
10
5
0
**
***
***
Control
BIC
J54
Combo
Relative c-PARP staining
30
20
10
0
***
***
Control
BIC
J54
Combo
Relative γH2AX staining
40
30
20
10
0
**
***
***
Control
BIC
J54
Combo
