# Supplementary material for: Generation of Phenothiazine with Potent Anti-TLK1 Activity for Prostate Cancer Therapy
Source: iScience. 2020 Aug 20;23(9):101474. doi: 10.1016/j.isci.2020.101474 (PMC7486443; doi:10.1016/j.isci.2020.101474)

^{1}H NMR spectra of J54

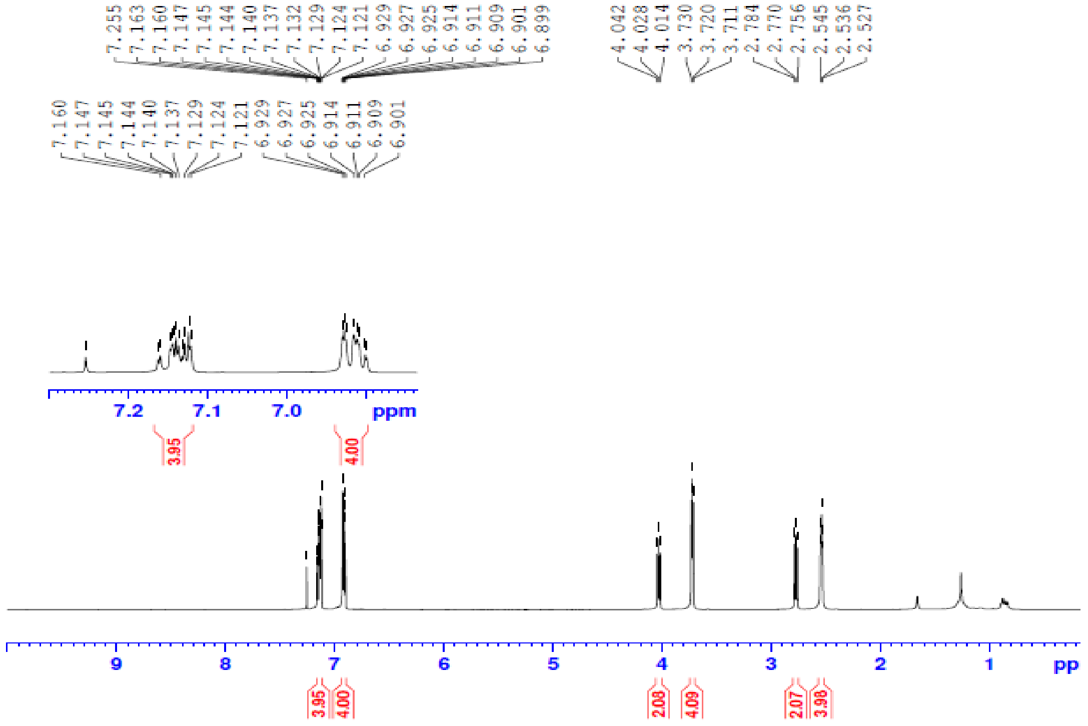

^{13}C NMR spectra of J54

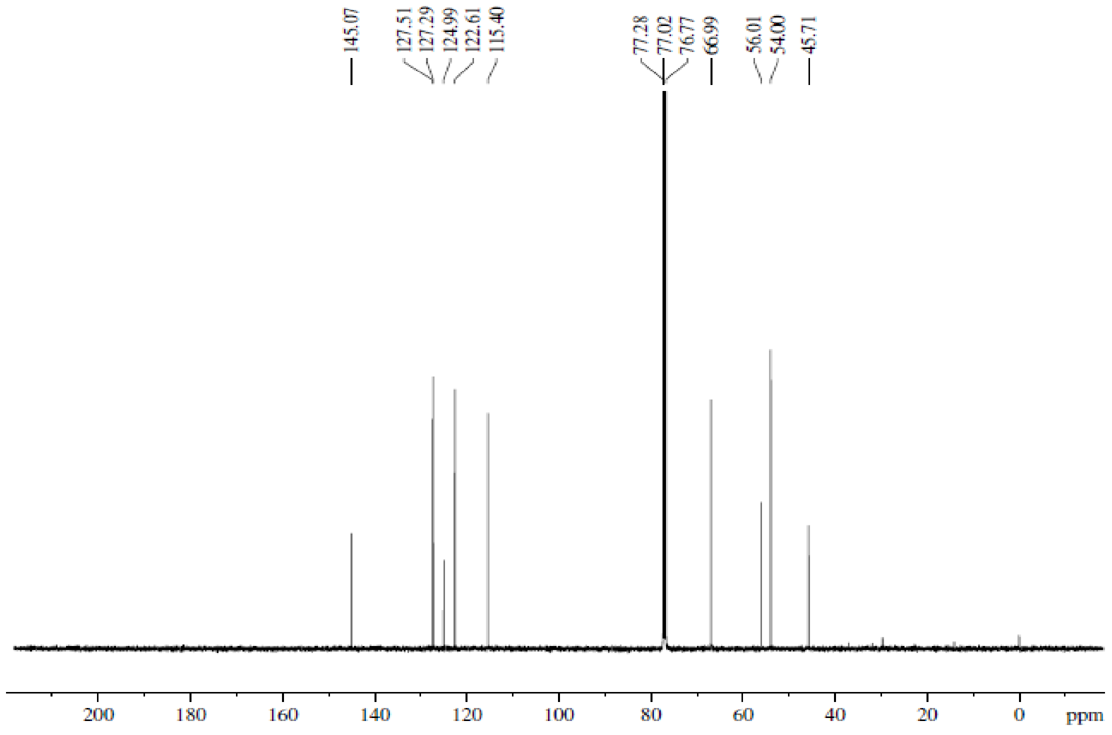

Mass Spectra of J54

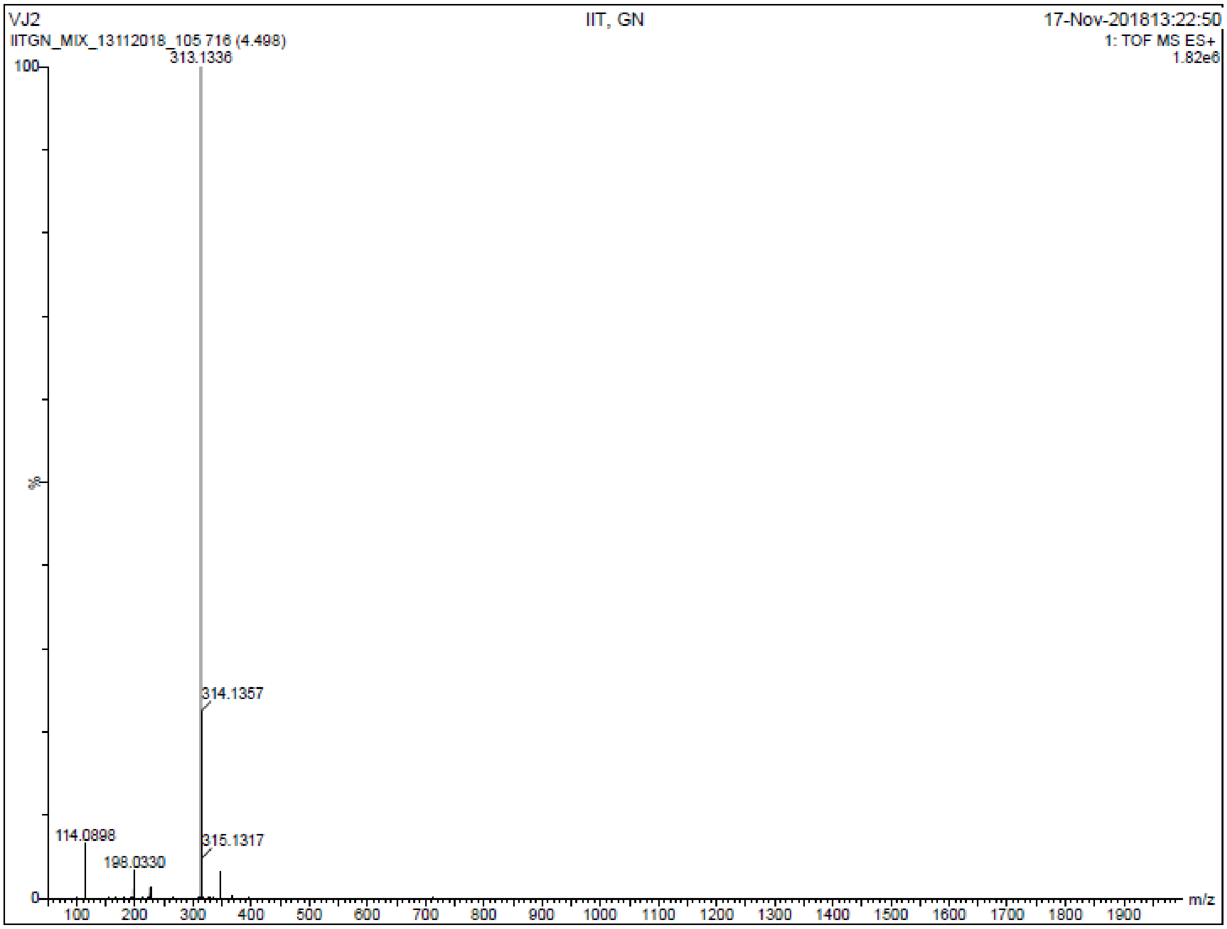

^{1}H NMR spectra of J54

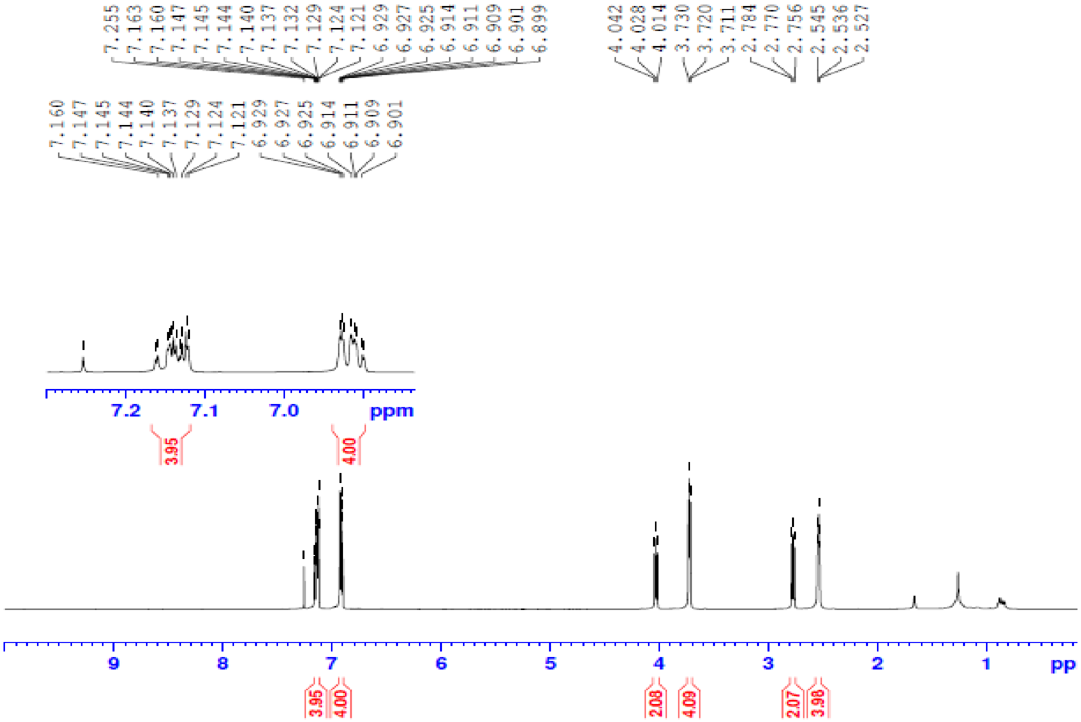

^{13}C NMR spectra of J54

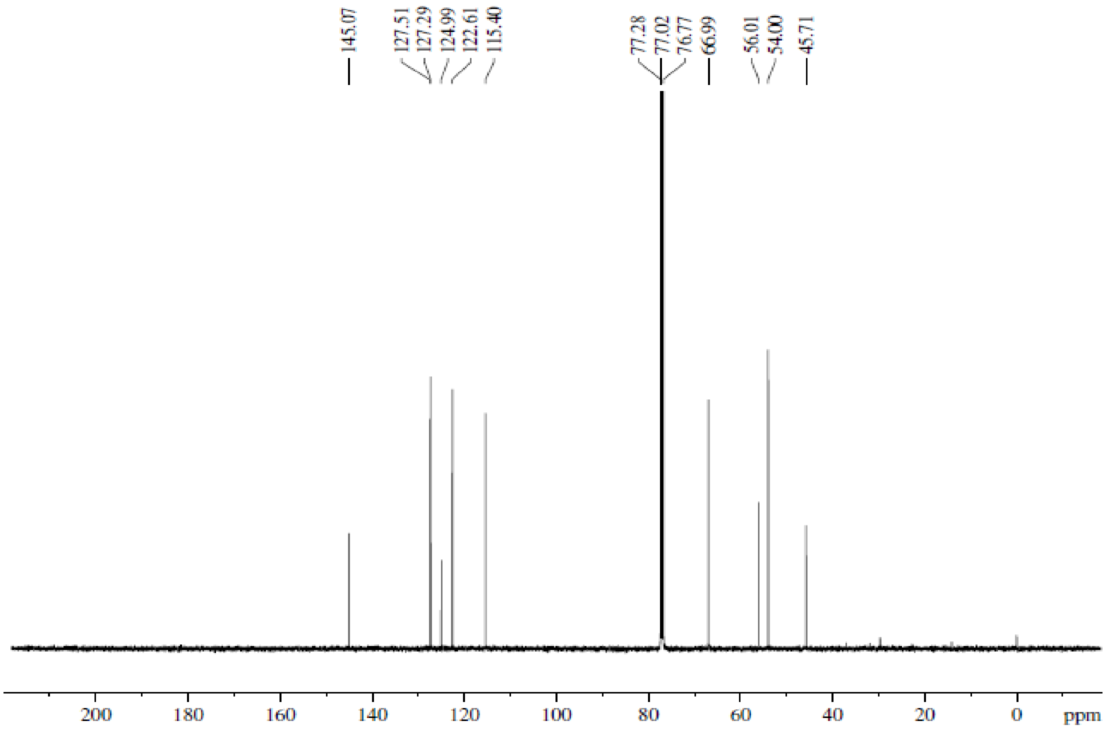

Mass Spectra of J54

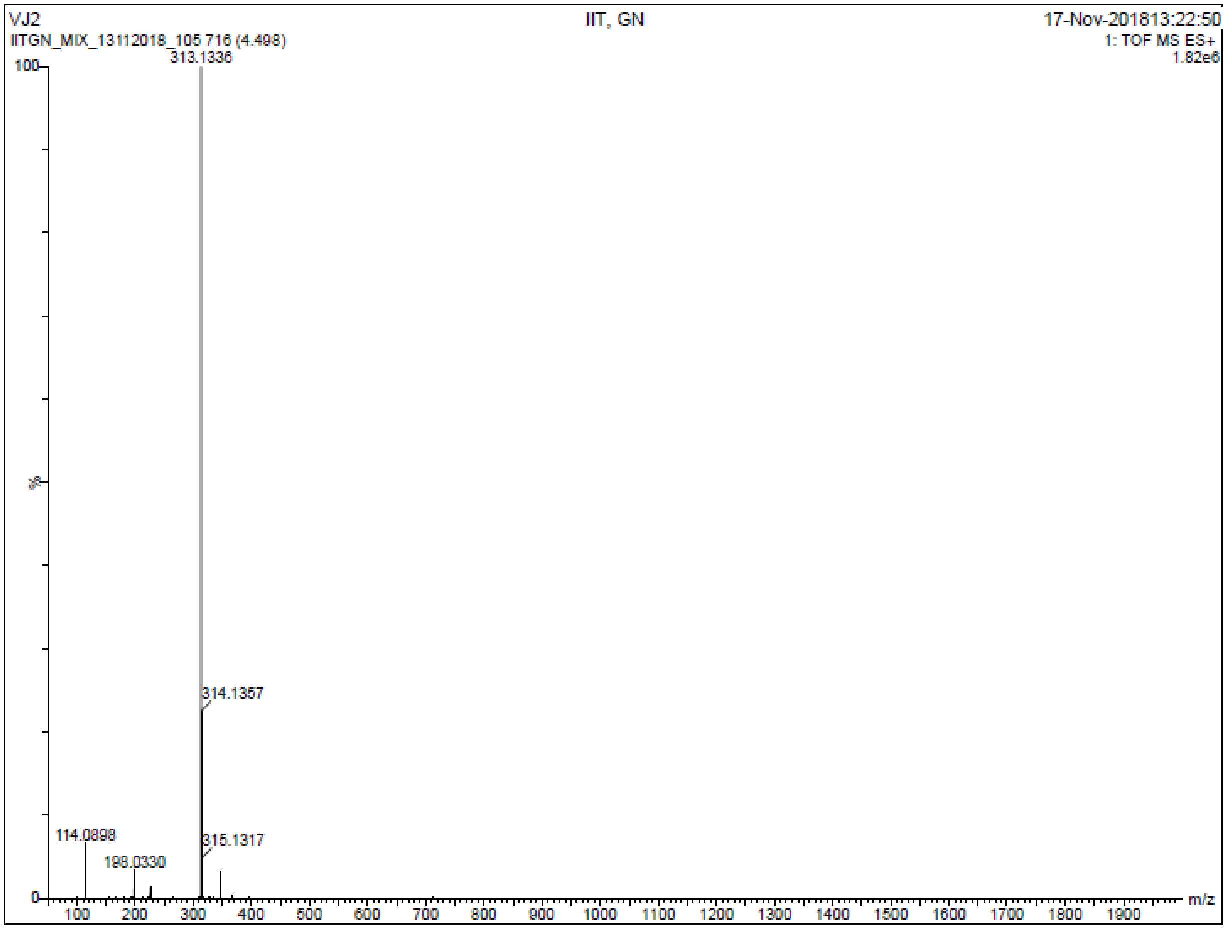

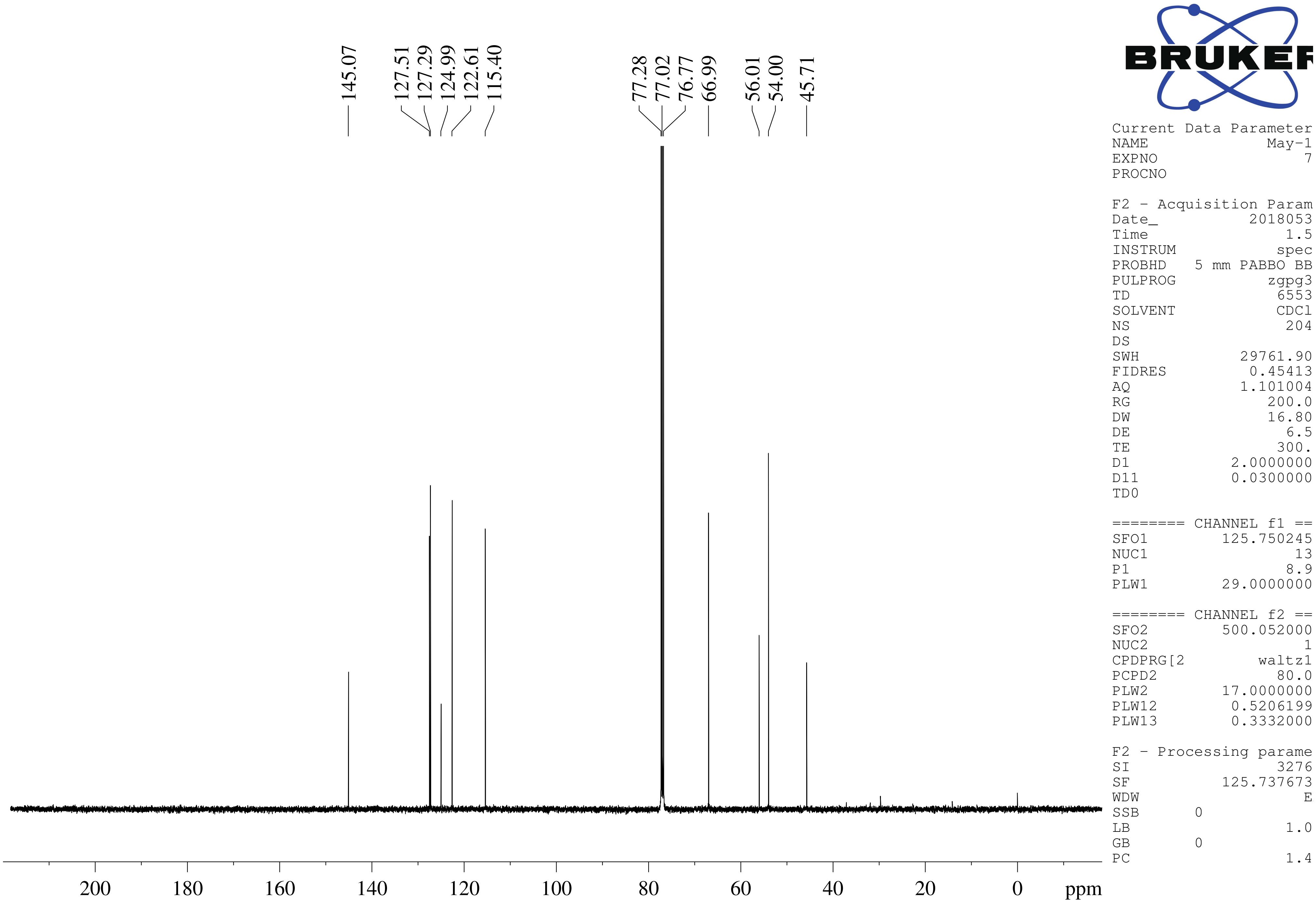
145.07
127.51
127.29
124.99
122.61
115.40
77.28
77.02
76.77
66.99
56.01
54.00
45.71
200
180
160
140
120
100
80
60
40
20
0
ppm
BRUKER
Current Data Parameter
NAME May-1
EXPNO 7
PROCNO
F2 - Acquisition Param
Date_ 2018053
Time 1.5
INSTRUM spec
PROBHD 5 mm PABBO BB
PULPROG zgpg3
TD 6553
SOLVENT CDCl
NS 204
DS
SWH 29761.90
FIDRES 0.45413
AQ 1.101004
RG 200.0
DW 16.80
DE 6.5
TE 300.
D1 2.0000000
D11 0.0300000
TD0
======== CHANNEL f1 ==
SFO1 125.750245
NUC1 13
P1 8.9
PLW1 29.0000000
======== CHANNEL f2 ==
SFO2 500.052000
NUC2 1
CPDPRG[2 waltz1
PCPD2 80.0
PLW2 17.0000000
PLW12 0.5206199
PLW13 0.3332000
F2 - Processing parame
SI 3276
SF 125.737673
WDW E
SSB 0
LB 1.0
GB 0
PC 1.4

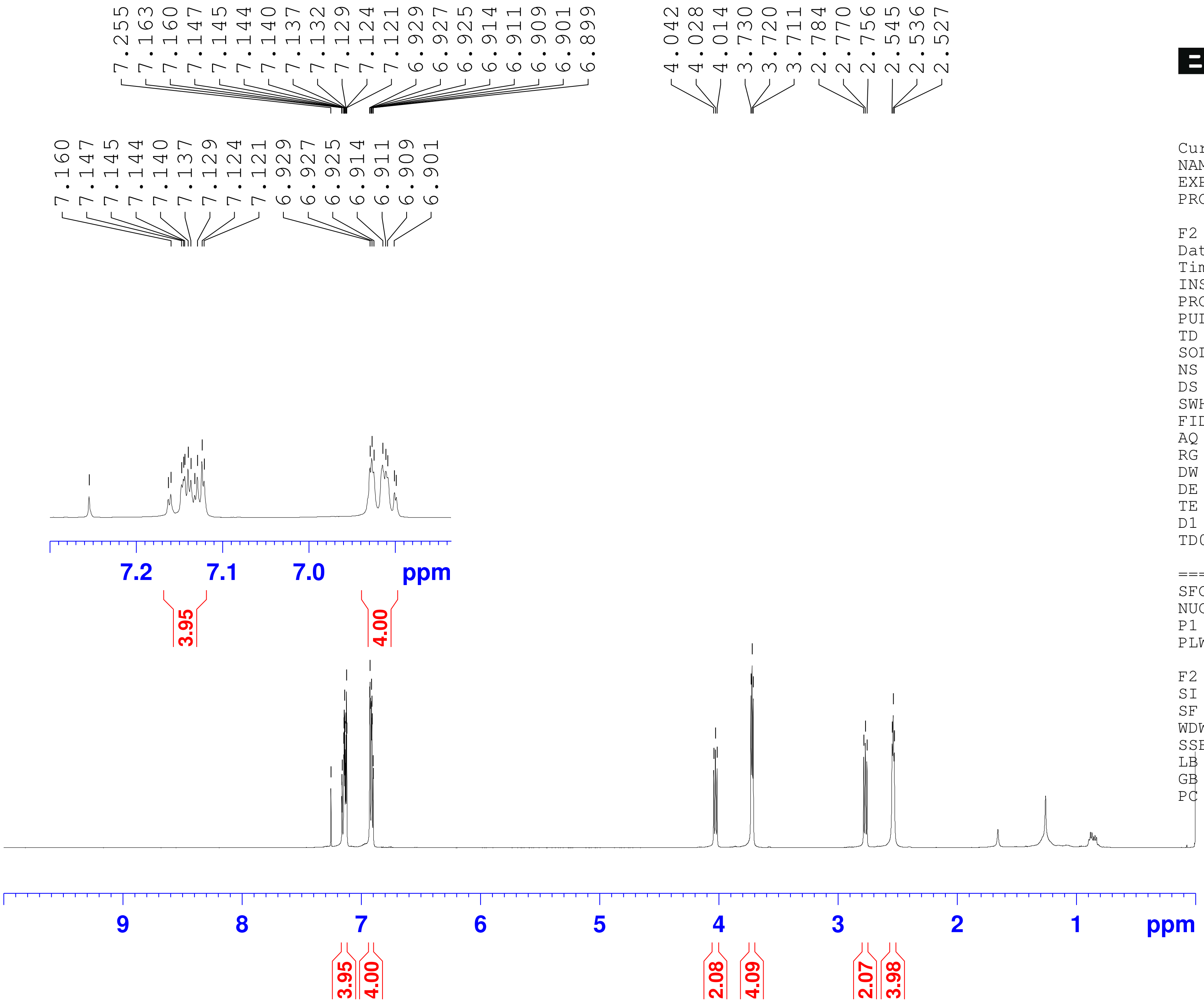

Current Data Parameters
NAME May-18
EXPNO 9
PROCNO 1

F2 - Acquisition Parameters
Date_ 20180510
Time 22.06
INSTRUM spect
PROBHD 5 mm PABBO BB/
PULPROG zg30
TD 65536
SOLVENT CDCl3
NS 32
DS 2
SWH 10000.000 Hz
FIDRES 0.152588 Hz
AQ 3.2767999 sec
RG 157.63
DW 50.000 usec
DE 6.50 usec
TE 300.0 K
D1 1.00000000 sec
TD0 1

======== CHANNEL f1 ========
SFO1 500.0530880 MHz
NUC1 1H
P1 12.15 usec
PLW1 17.00000000 W

F2 - Processing parameters
SI 65536
SF 500.0500197 MHz
WDW EM
SSB 0
LB 0.30 Hz
GB 0
PC 1.00

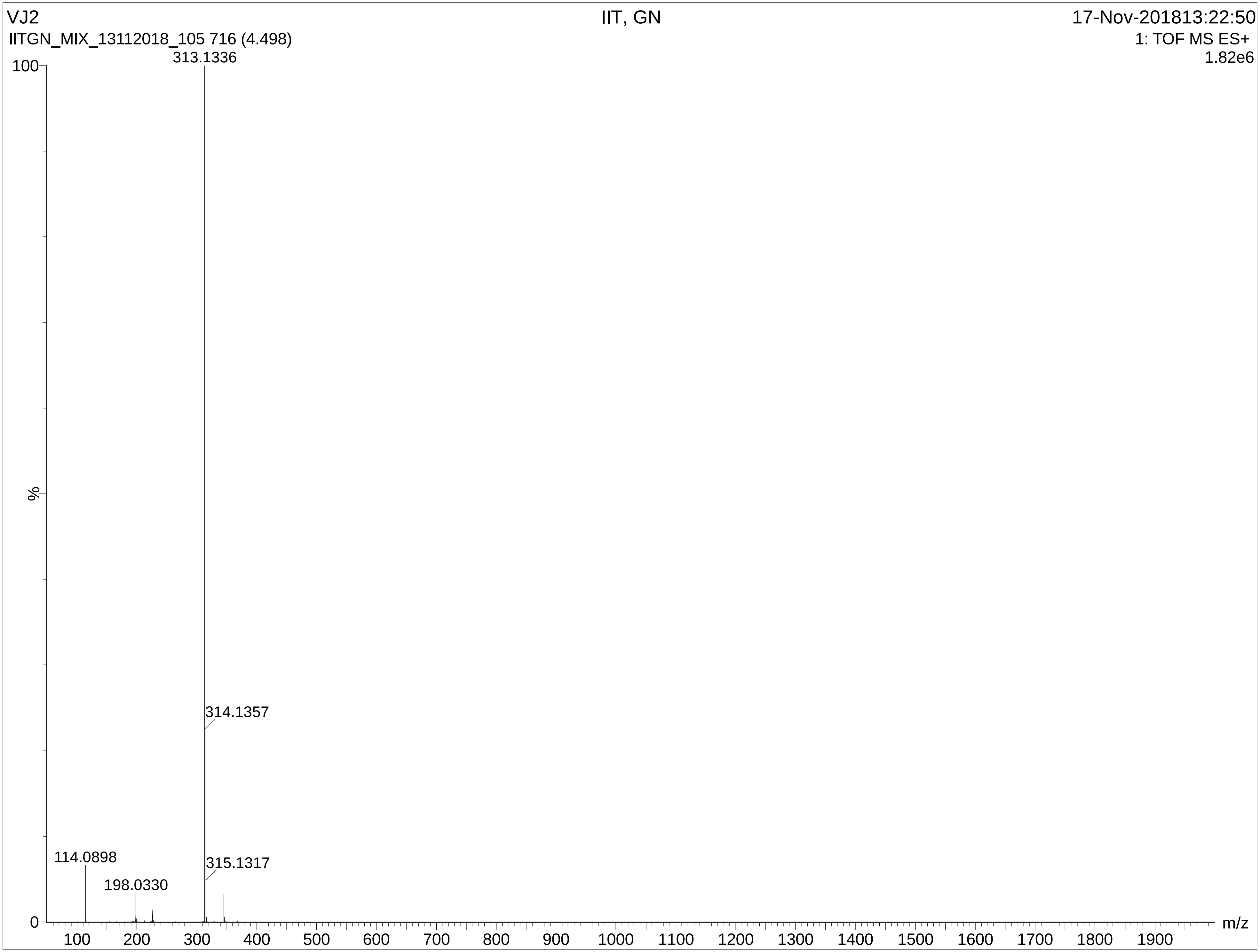

VJ2
IIT, GN
17-Nov-201813:22:50
IITGN_MIX_13112018_105 716 (4.498)
1: TOF MS ES+
1.82e6
313.1336
314.1357
315.1317
114.0898
198.0330
100
%
0
m/z
100
200
300
400
500
600
700
800
900
1000
1100
1200
1300
1400
1500
1600
1700
1800
1900

checkCIF/PLATON report

Structure factors have been supplied for datablock(s) 2_moxp_lrt

THIS REPORT IS FOR GUIDANCE ONLY. IF USED AS PART OF A REVIEW PROCEDURE FOR PUBLICATION, IT SHOULD NOT REPLACE THE EXPERTISE OF AN EXPERIENCED CRYSTALLOGRAPHIC REFEREE.

No syntax errors found. CIF dictionary Interpreting this report

Datablock: 2_moxp_lrt

Bond precision: C-C = 0.0067 A Wavelength=1.54184

Cell:	a=9.6623(8)	b=8.7108(7)	c=19.3352(18)
	alpha=90	beta=102.717(9)	gamma=90
Temperature:	150 K		

	Calculated	Reported
Volume	1587.5(2)	1587.5(2)
Space group	P 21/c	P 1 21/c 1
Hall group	-P 2ybc	-P 2ybc
Moiety formula	C18 H20 N2 O S	C18 H20 N2 O S
Sum formula	C18 H20 N2 O S	C18 H20 N2 O S
Mr	312.42	312.42
Dx,g cm-3	1.307	1.307
Z	4	4
Mu (mm-1)	1.827	1.827
F000	664.0	664.0
F000’	666.98	
h,k,lmax	11,10,23	11,10,23
Nref	2827	2728
Tmin,Tmax	0.600,0.870	0.584,1.000
Tmin’	0.496	

Correction method= # Reported T Limits: Tmin=0.584 Tmax=1.000
AbsCorr = MULTI-SCAN

Data completeness= 0.965 Theta(max)= 66.768

R(reflections)= 0.0755(1921) wR2(reflections)= 0.3024(2728)

S = 1.146 Npar= 199

The following ALERTS were generated. Each ALERT has the format
test-name_ALERT_alert-type_alert-level.
Click on the hyperlinks for more details of the test.

Alert level C

```
PLAT029_ALERT_3_C _diffrn_measured_fraction_theta_full value Low .      0.965 Why?
PLAT084_ALERT_3_C High wR2 Value (i.e. > 0.25) ...................       0.30 Report
PLAT340_ALERT_3_C Low Bond Precision on  C-C Bonds ...............    0.00667 Ang.
PLAT906_ALERT_3_C Large K Value in the Analysis of Variance ......      2.674 Check
PLAT911_ALERT_3_C Missing FCF Refl Between Thmin & STh/L=    0.596        100 Report
PLAT977_ALERT_2_C Check Negative Difference Density on H14B             -0.40 eA-3
```

Alert level G

```
PLAT072_ALERT_2_G SHELXL First  Parameter in WGHT  Unusually Large       0.20 Report
PLAT398_ALERT_2_G Deviating  C-O-C    Angle From 120 for O1             109.5 Degree
PLAT909_ALERT_3_G Percentage of I>2sig(I) Data at Theta(Max) Still        63% Note
PLAT941_ALERT_3_G Average HKL Measurement Multiplicity ...........        1.7 Low
PLAT955_ALERT_1_G Reported (CIF) and Actual (FCF) Lmax Differ by .          1 Units
PLAT978_ALERT_2_G Number C-C Bonds with Positive Residual Density.          2 Info
PLAT992_ALERT_5_G Repd & Actual _reflns_number_gt Values Differ by          1 Check
```

---

0 **ALERT level A** = Most likely a serious problem - resolve or explain
0 **ALERT level B** = A potentially serious problem, consider carefully
6 **ALERT level C** = Check. Ensure it is not caused by an omission or oversight
7 **ALERT level G** = General information/check it is not something unexpected

1 ALERT type 1 CIF construction/syntax error, inconsistent or missing data
4 ALERT type 2 Indicator that the structure model may be wrong or deficient
7 ALERT type 3 Indicator that the structure quality may be low
0 ALERT type 4 Improvement, methodology, query or suggestion
1 ALERT type 5 Informative message, check

Validation response form

Please find below a validation response form (VRF) that can be filled in and pasted into your CIF.

```
# start Validation Reply Form
_vrf_PLAT029_2_moxp_lrt
;
PROBLEM: _diffrn_measured_fraction_theta_full value Low .      0.965 Why?
RESPONSE: ...
;
_vrf_PLAT084_2_moxp_lrt
;
PROBLEM: High wR2 Value (i.e. > 0.25) ...................       0.30 Report
RESPONSE: ...
;
_vrf_PLAT340_2_moxp_lrt
;
PROBLEM: Low Bond Precision on  C-C Bonds ...............    0.00667 Ang.
RESPONSE: ...
;
_vrf_PLAT906_2_moxp_lrt
;
PROBLEM: Large K Value in the Analysis of Variance ......      2.674 Check
RESPONSE: ...
;
_vrf_PLAT911_2_moxp_lrt
;
PROBLEM: Missing FCF Refl Between Thmin & STh/L=    0.596        100 Report
RESPONSE: ...
;
```

```
_vrf_PLAT977_2_moxp_lrt
;
PROBLEM: Check Negative Difference Density on H14B             -0.40 eA-3
RESPONSE: ...
;
# end Validation Reply Form
```

It is advisable to attempt to resolve as many as possible of the alerts in all categories. Often the minor alerts point to easily fixed oversights, errors and omissions in your CIF or refinement strategy, so attention to these fine details can be worthwhile. In order to resolve some of the more serious problems it may be necessary to carry out additional measurements or structure refinements. However, the purpose of your study may justify the reported deviations and the more serious of these should normally be commented upon in the discussion or experimental section of a paper or in the "special_details" fields of the CIF. checkCIF was carefully designed to identify outliers and unusual parameters, but every test has its limitations and alerts that are not important in a particular case may appear. Conversely, the absence of alerts does not guarantee there are no aspects of the results needing attention. It is up to the individual to critically assess their own results and, if necessary, seek expert advice.

Publication of your CIF in IUCr journals

A basic structural check has been run on your CIF. These basic checks will be run on all CIFs submitted for publication in IUCr journals (*Acta Crystallographica*, *Journal of Applied Crystallography*, *Journal of Synchrotron Radiation*); however, if you intend to submit to *Acta Crystallographica Section C* or *E* or *IUCrData*, you should make sure that full publication checks are run on the final version of your CIF prior to submission.

Publication of your CIF in other journals

Please refer to the *Notes for Authors* of the relevant journal for any special instructions relating to CIF submission.

PLATON version of 22/04/2020; check.def file version of 09/03/2020

Datablock 2_moxp_lrt - ellipsoid plot

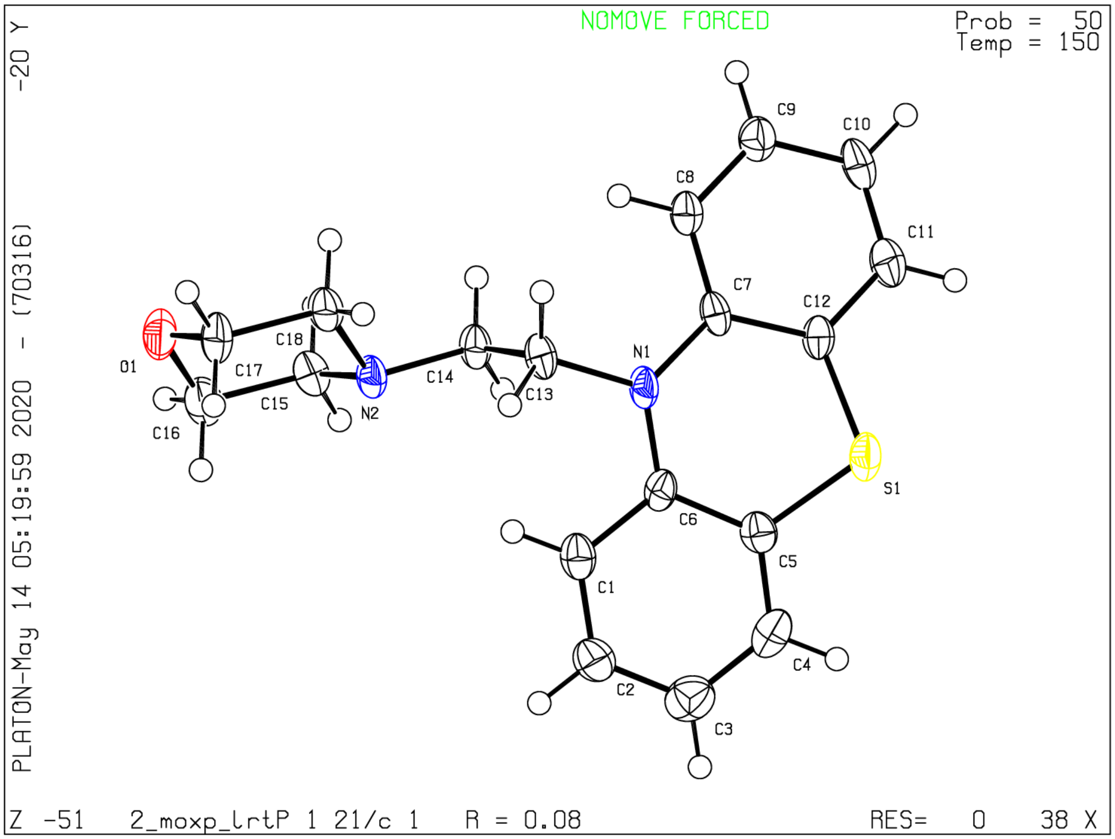

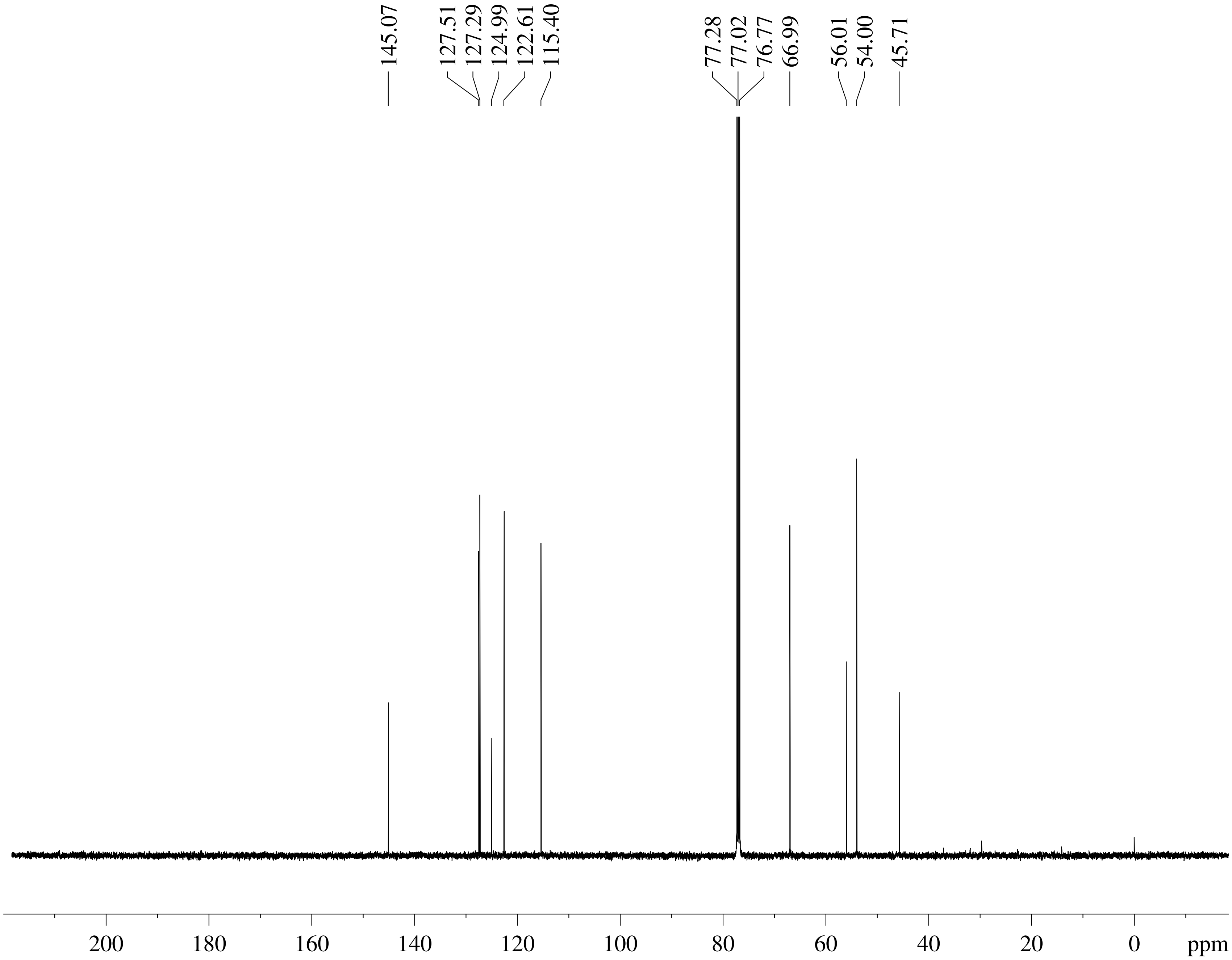

Current Data Parameter
NAME May-1
EXPNO 7
PROCNO

F2 - Acquisition Param
Date_ 2018053
Time 1.5
INSTRUM spec
PROBHD 5 mm PABBO BB
PULPROG zgpg3
TD 6553
SOLVENT CDCl
NS 204
DS
SWH 29761.90
FIDRES 0.45413
AQ 1.101004
RG 200.0
DW 16.80
DE 6.5
TE 300.
D1 2.0000000
D11 0.0300000
TD0

======== CHANNEL f1 ==
SFO1 125.750245
NUC1 13
P1 8.9
PLW1 29.0000000

======== CHANNEL f2 ==
SFO2 500.052000
NUC2 1
CPDPRG[2 waltz1
PCPD2 80.0
PLW2 17.0000000
PLW12 0.5206199
PLW13 0.3332000

F2 - Processing parame
SI 3276
SF 125.737673
WDW E
SSB 0
LB 1.0
GB 0
PC 1.4

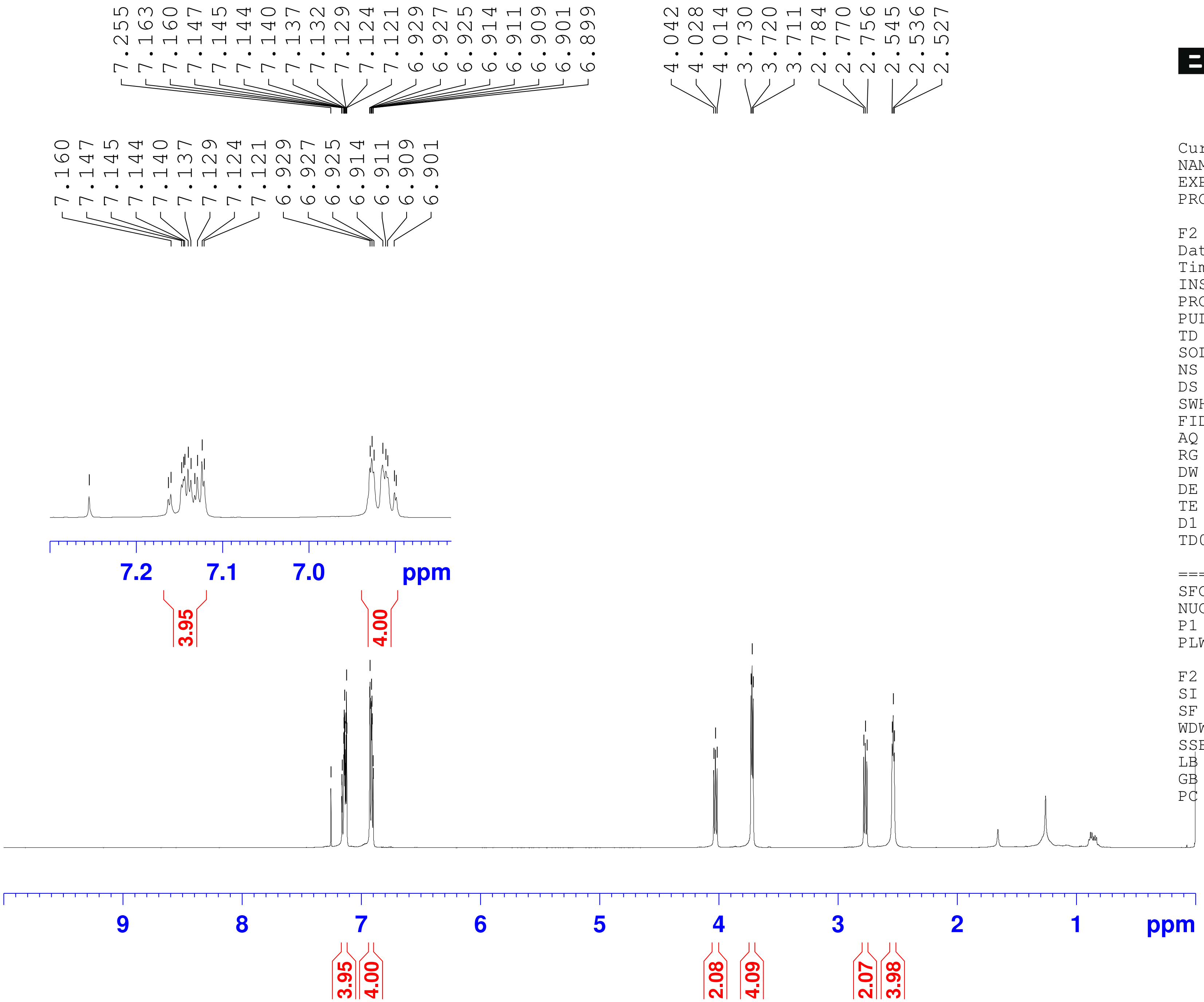

BRUKER

Current Data Parameters
NAME May-18
EXPNO 9
PROCNO 1

F2 - Acquisition Parameters
Date_ 20180510
Time 22.06
INSTRUM spect
PROBHD 5 mm PABBO BB/
PULPROG zg30
TD 65536
SOLVENT CDCl3
NS 32
DS 2
SWH 10000.000 Hz
FIDRES 0.152588 Hz
AQ 3.2767999 sec
RG 157.63
DW 50.000 usec
DE 6.50 usec
TE 300.0 K
D1 1.00000000 sec
TD0 1

======== CHANNEL f1 ========
SFO1 500.0530880 MHz
NUC1 1H
P1 12.15 usec
PLW1 17.00000000 W

F2 - Processing parameters
SI 65536
SF 500.0500197 MHz
WDW EM
SSB 0
LB 0.30 Hz
GB 0
PC 1.00

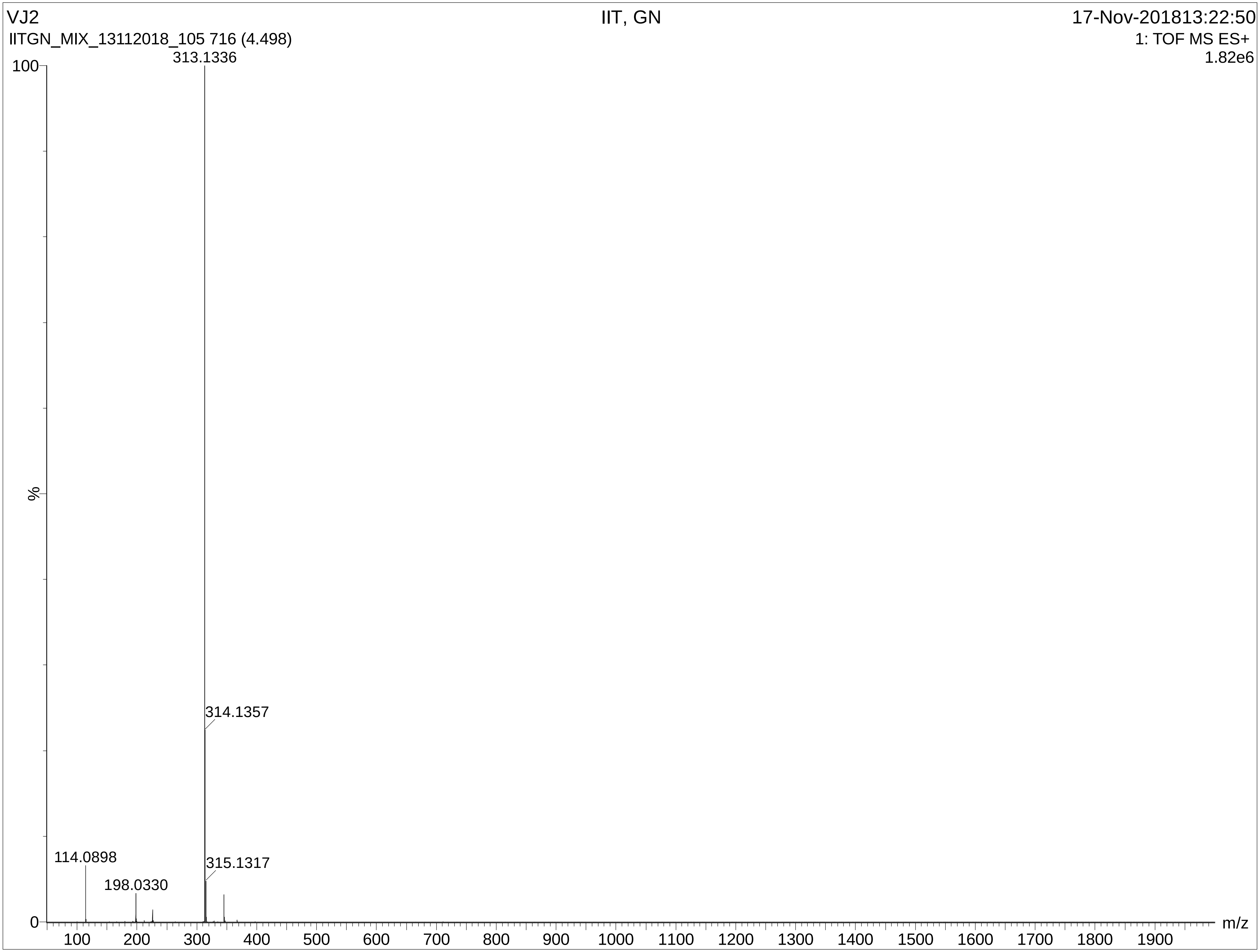

VJ2
IIT, GN
17-Nov-201813:22:50
IITGN_MIX_13112018_105 716 (4.498)
1: TOF MS ES+
1.82e6
313.1336
314.1357
114.0898
198.0330
315.1317
100
%
0
100
200
300
400
500
600
700
800
900
1000
1100
1200
1300
1400
1500
1600
1700
1800
1900
m/z

checkCIF/PLATON report

Structure factors have been supplied for datablock(s) 2_moxp_lrt

THIS REPORT IS FOR GUIDANCE ONLY. IF USED AS PART OF A REVIEW PROCEDURE FOR PUBLICATION, IT SHOULD NOT REPLACE THE EXPERTISE OF AN EXPERIENCED CRYSTALLOGRAPHIC REFEREE.

No syntax errors found.   CIF dictionary   Interpreting this report

Datablock: 2_moxp_lrt

```
Bond precision:  C-C = 0.0067 A          Wavelength=1.54184

Cell:           a=9.6623(8)    b=8.7108(7)         c=19.3352(18)
                alpha=90       beta=102.717(9)     gamma=90
Temperature:    150 K

                Calculated                 Reported
Volume          1587.5(2)                  1587.5(2)
Space group     P 21/c                     P 1 21/c 1
Hall group      -P 2ybc                    -P 2ybc
Moiety formula  C18 H20 N2 O S             C18 H20 N2 O S
Sum formula     C18 H20 N2 O S             C18 H20 N2 O S
Mr              312.42                     312.42
Dx,g cm-3       1.307                      1.307
Z               4                          4
Mu (mm-1)       1.827                      1.827
F000            664.0                      664.0
F000'           666.98
h,k,lmax        11,10,23                   11,10,23
Nref            2827                       2728
Tmin,Tmax       0.600,0.870                0.584,1.000
Tmin'           0.496

Correction method= # Reported T Limits: Tmin=0.584 Tmax=1.000
AbsCorr = MULTI-SCAN

Data completeness= 0.965          Theta(max)= 66.768

R(reflections)= 0.0755( 1921)     wR2(reflections)= 0.3024( 2728)

S = 1.146                  Npar= 199
```

The following ALERTS were generated. Each ALERT has the format
test-name_ALERT_alert-type_alert-level.
Click on the hyperlinks for more details of the test.

Alert level C

```
PLAT029_ALERT_3_C _diffrn_measured_fraction_theta_full value Low .      0.965 Why?
PLAT084_ALERT_3_C High wR2 Value (i.e. > 0.25) ...................       0.30 Report
PLAT340_ALERT_3_C Low Bond Precision on  C-C Bonds ...............    0.00667 Ang.
PLAT906_ALERT_3_C Large K Value in the Analysis of Variance ......      2.674 Check
PLAT911_ALERT_3_C Missing FCF Refl Between Thmin & STh/L=    0.596        100 Report
PLAT977_ALERT_2_C Check Negative Difference Density on H14B             -0.40 eA-3
```

Alert level G

```
PLAT072_ALERT_2_G SHELXL First  Parameter in WGHT  Unusually Large       0.20 Report
PLAT398_ALERT_2_G Deviating  C-O-C    Angle From 120 for O1             109.5 Degree
PLAT909_ALERT_3_G Percentage of I>2sig(I) Data at Theta(Max) Still        63% Note
PLAT941_ALERT_3_G Average HKL Measurement Multiplicity ...........        1.7 Low
PLAT955_ALERT_1_G Reported (CIF) and Actual (FCF) Lmax Differ by .          1 Units
PLAT978_ALERT_2_G Number C-C Bonds with Positive Residual Density.          2 Info
PLAT992_ALERT_5_G Repd & Actual _reflns_number_gt Values Differ by          1 Check
```

```
   0 ALERT level A = Most likely a serious problem - resolve or explain
   0 ALERT level B = A potentially serious problem, consider carefully
   6 ALERT level C = Check. Ensure it is not caused by an omission or oversight
   7 ALERT level G = General information/check it is not something unexpected

   1 ALERT type 1 CIF construction/syntax error, inconsistent or missing data
   4 ALERT type 2 Indicator that the structure model may be wrong or deficient
   7 ALERT type 3 Indicator that the structure quality may be low
   0 ALERT type 4 Improvement, methodology, query or suggestion
   1 ALERT type 5 Informative message, check
```

Validation response form

Please find below a validation response form (VRF) that can be filled in and pasted into your CIF.

```
# start Validation Reply Form
_vrf_PLAT029_2_moxp_lrt
;
PROBLEM: _diffrn_measured_fraction_theta_full value Low .      0.965 Why?
RESPONSE: ...
;
_vrf_PLAT084_2_moxp_lrt
;
PROBLEM: High wR2 Value (i.e. > 0.25) ...................       0.30 Report
RESPONSE: ...
;
_vrf_PLAT340_2_moxp_lrt
;
PROBLEM: Low Bond Precision on  C-C Bonds ...............    0.00667 Ang.
RESPONSE: ...
;
_vrf_PLAT906_2_moxp_lrt
;
PROBLEM: Large K Value in the Analysis of Variance ......      2.674 Check
RESPONSE: ...
;
_vrf_PLAT911_2_moxp_lrt
;
PROBLEM: Missing FCF Refl Between Thmin & STh/L=    0.596        100 Report
RESPONSE: ...
;
```

```
_vrf_PLAT977_2_moxp_lrt
;
PROBLEM: Check Negative Difference Density on H14B            -0.40 eA-3
RESPONSE: ...
;
# end Validation Reply Form
```

It is advisable to attempt to resolve as many as possible of the alerts in all categories. Often the minor alerts point to easily fixed oversights, errors and omissions in your CIF or refinement strategy, so attention to these fine details can be worthwhile. In order to resolve some of the more serious problems it may be necessary to carry out additional measurements or structure refinements. However, the purpose of your study may justify the reported deviations and the more serious of these should normally be commented upon in the discussion or experimental section of a paper or in the "special_details" fields of the CIF. checkCIF was carefully designed to identify outliers and unusual parameters, but every test has its limitations and alerts that are not important in a particular case may appear. Conversely, the absence of alerts does not guarantee there are no aspects of the results needing attention. It is up to the individual to critically assess their own results and, if necessary, seek expert advice.

Publication of your CIF in IUCr journals

A basic structural check has been run on your CIF. These basic checks will be run on all CIFs submitted for publication in IUCr journals (*Acta Crystallographica*, *Journal of Applied Crystallography*, *Journal of Synchrotron Radiation*); however, if you intend to submit to *Acta Crystallographica Section C* or *E* or *IUCrData*, you should make sure that full publication checks are run on the final version of your CIF prior to submission.

Publication of your CIF in other journals

Please refer to the *Notes for Authors* of the relevant journal for any special instructions relating to CIF submission.

PLATON version of 22/04/2020; check.def file version of 09/03/2020

Datablock 2_moxp_lrt - ellipsoid plot

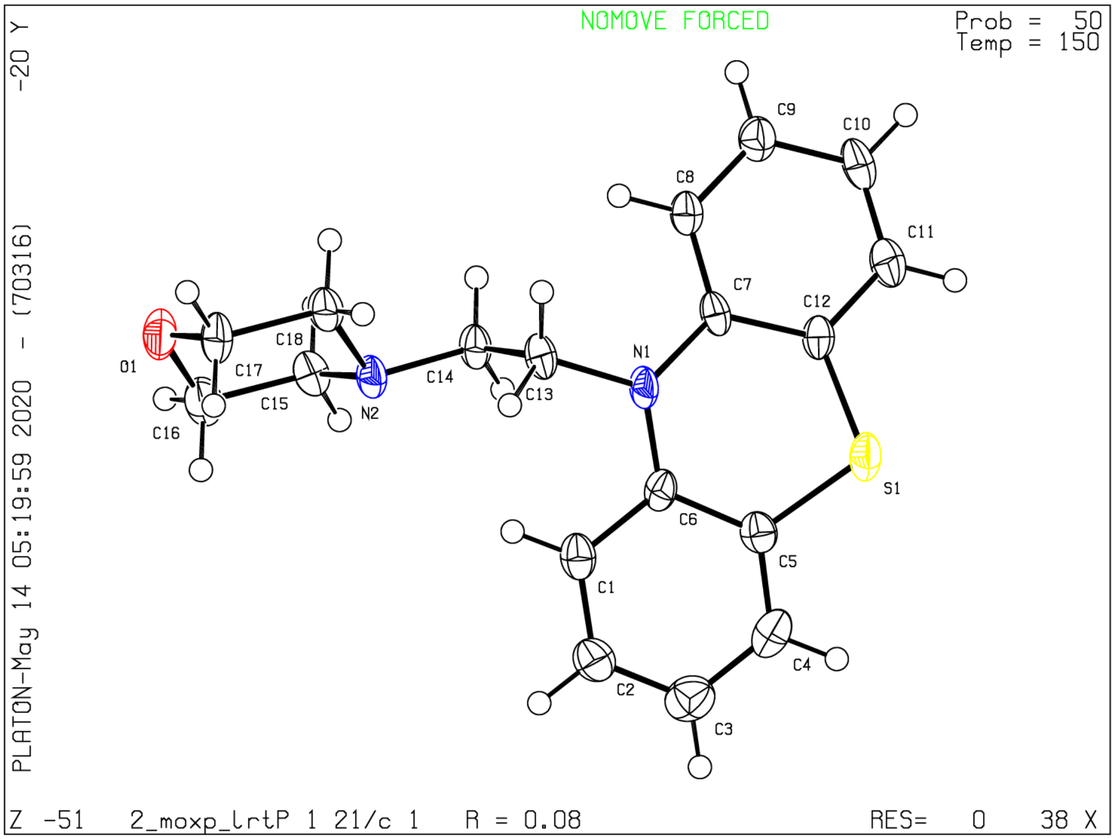

Supplement: Data S2. Binder Spectral Data, Related to Figure 1 — Characterization of J54 and J56. [file mmc4.pdf]
